# One hundred years of excellence: the top one hundred authors of the Journal of Comparative Physiology A

**DOI:** 10.1007/s00359-024-01699-1

**Published:** 2024-03-29

**Authors:** Günther K. H. Zupanc, Uwe Homberg, Charlotte Helfrich-Förster, Eric J. Warrant, Andrea Megela Simmons

**Affiliations:** 1 Department of Biology, Northeastern University, Boston, MA 02115 USA; 2 Department of Biology, Philipps-University of Marburg, 35032 Marburg, Germany; 3 Neurobiology and Genetics, Biocentre, University of Würzburg, 97074 Würzburg, Germany; 4 Department of Biology, University of Lund, 22362 Lund, Sweden; 5 Department of Cognitive, Linguistic and Psychological Sciences, Brown University, Providence, RI 02912 USA

**Keywords:** Journal of Comparative Physiology A, Zeitschrift für vergleichende Physiologie, Karl von Frisch, Hansjochem Autrum, Women in Science

## Abstract

The *Journal of Comparative Physiology A* is the premier peer-reviewed scientific journal in comparative physiology, in particular sensory physiology, neurophysiology, and neuroethology. Founded in 1924 by Karl von Frisch and Alfred Kühn, it celebrates its 100th anniversary in 2024. During these 100 years, many of the landmark achievements in these disciplines were published in this journal. To commemorate these accomplishments, we have compiled a list of the Top 100 Authors over these 100 years, representing approximately 1% of all its authors. To select these individuals, three performance criteria were applied: number of publications, total number of citations attracted by these articles, and mean citation rate of the papers published by each author. The resulting list of the Top 100 Authors provides a fascinating insight into the history of the disciplines covered by the *Journal of Comparative Physiology A* and into the academic careers of many of their leading representatives.

## Introduction

**Fig. 1:**
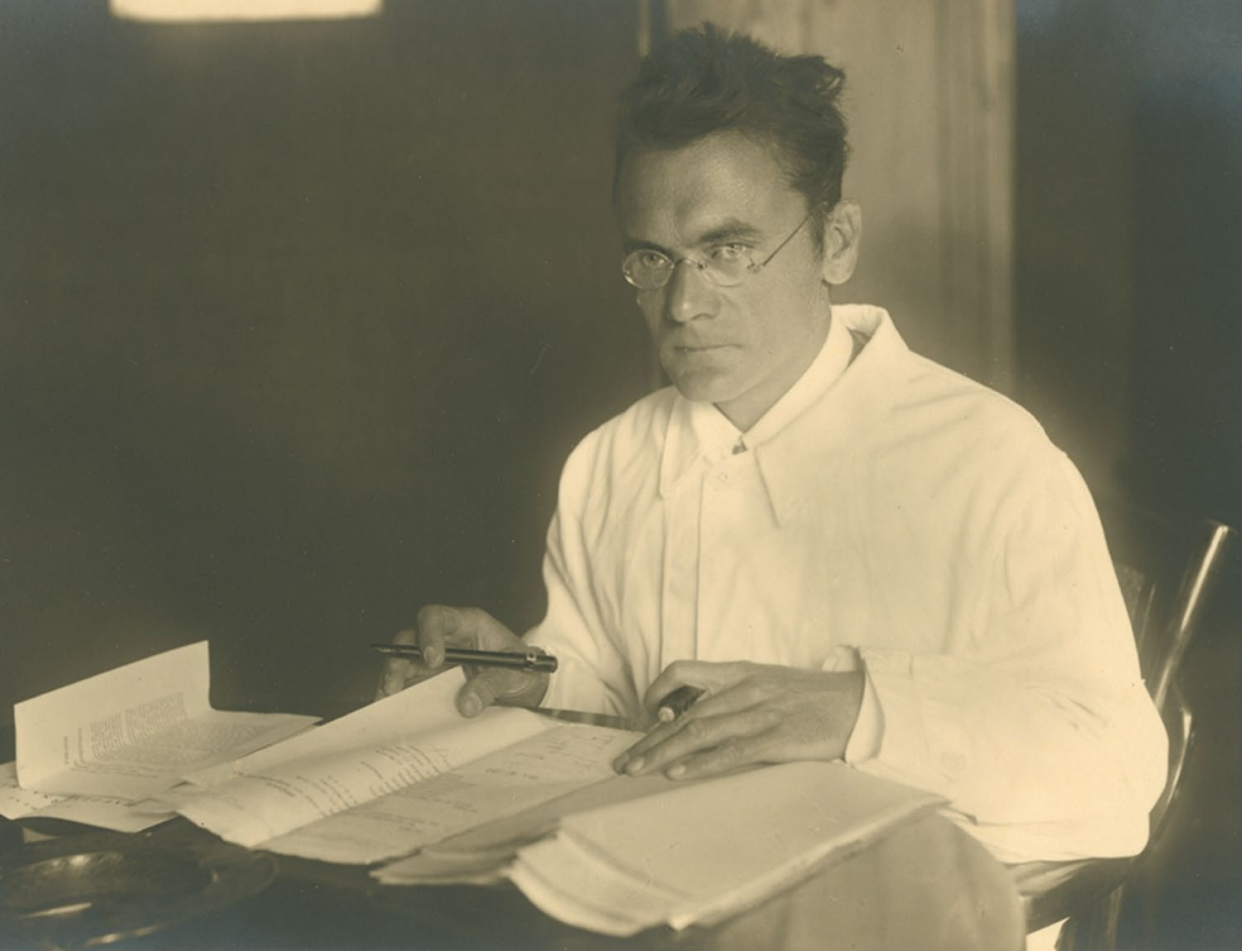
Karl von Frisch. He was co-founder of what later became the Journal of Comparative Physiology A and served as its editor-in-chief from 1924 to 1960. The photograph was taken by R. Obernetter (according to handwritten notes on the back of a print) in Rostock or Breslau. Karl von Frisch was *Ordinarius* (full professor) in Rostock from 1921 to 1923, and in Breslau from 1923 to 1925. Although the exact date of the photo is unknown, the information about the location suggests that it was taken between 1921 and 1925. Courtesy: Bayerische Staatsbibliothek München/Bildarchiv

The *Journal of Comparative Physiology A* was founded by Karl von Frisch (Fig. 1) and Alfred Kühn under its German title *Zeitschrift für vergleichende Physiologie* (for a historical account see Zupanc 2022). Its first two issues appeared in March 1924. For the first twelve years, it was published as Section C of the *Zeitschrift für wissenschaftliche Biologie*. Karl von Frisch remained its editor-in-chief until 1960, when Hansjochem Autrum succeeded him in this role. Like von Frisch, he was chief editor for 36 years. In 1996, Autrum transferred the overall responsibility for the *Journal* to Friedrich G. Barth. By the end of 2021, Barth retired from this position, and then one of us (GKHZ) became the fourth editor-in-chief of the *Journal of Comparative Physiology A*.

Under Autrum, the journal had attracted increasing numbers of submissions from authors outside Germany, and finally articles were published in English only. The global orientation was formalized by changing its name to *Journal of Comparative Physiology* in 1972. In line with the rising trend toward specialization in science, it split into two daughter journals four years later, covering sensory physiology, neurophysiology, and neuroethology in the *Journal of Comparative Physiology A*, and biochemical, systemic, and environmental physiology in the *Journal of Comparative Physiology B*. For the sake of simplicity, in the following we will refer to the two sister journals and their two predecessors by ‘JCPA’ and ‘JCPB,’ respectively.

As the first journal dedicated to publishing research in comparative physiology, JCPA provided a prominent platform for propelling the development of this new discipline. As a spin-off of this process, JCPA played an important role in the emergence of neuroethology. Under the leadership of von Frisch and Autrum, it became *the* leading journal in comparative physiology and neuroethology. However, since the early 2000s, JCPA has faced increasing competition from open-access journals. It has responded to this challenge by implementing an open-access option within its traditional subscription business model. Despite all these changes over the 100 years of JCPA’s existence, what has remained a steady force behind its mission is the determination of its editors to catalyze the production of articles of highest quality. This includes fair and constructive peer review, and comprehensive support throughout the editorial handling of submitted manuscripts. However, the production of high-quality articles is only possible as long as scientists trust the editors with their finest pieces of research. A look at the list of authors who have published in JCPA over the last 100 years quickly supports this claim.

To celebrate the outstanding contributions of the many members of the community to JCPA, we have compiled a list of the Top 100 Authors of the Journal over the last 100 years, representing approximately 1% of all authors. To select these individuals, we applied three performance criteria, without claiming that the contributions of authors who are not included in this list are less valuable for the advances in comparative physiology and neuroethology than those of the Top 100 Authors. This list not only provides a fascinating insight into the history of these disciplines but also reminds us of the decades-long failure of society as a whole, and the scientific community in particular, to support women in science.

## Performance criteria and data analysis

The Top 100 Authors of JCPA during the first 100 years of its existence were determined by three performance criteria:

(i) Number of articles published in JCPA.
(ii) Total number of citations that these articles attracted. This number is widely used as an indicator of the peer recognition of an author. It correlates with the total number of papers an author published in JCPA.
(iii) Mean citations per article. This number reflects the impact that the articles published in JCPA by an author have on the work of other scientists and is independent of the number of publications.

For this analysis, all 9445 articles published in JCPA by December 31, 2023 were analyzed, based on data collected using the Dimensions platform (Digital Science & Research Solutions, Inc., London, U.K.), available at http://app.dimensions.ai.

According to this database, 8083 authors published in JCPA. The vast majority of them (5004) authored one article. Two or three articles were published by 1427 and 608 authors, respectively. Two-hundred-thirty-seven authors published 10 or more articles.

The total number of authors may, to some unknown extent, be inflated, as individuals who published under different names were counted as different authors. For example, Hansjochem Autrum published 23 articles under his full name and 7 articles under ‘H. Autrum’. Similarly, in a few cases the algorithm used by the Dimensions platform incorrectly attributed articles published by the same author to different authors because of different institutional affiliations under which these articles were published. In all such cases that we discovered, the errors were manually corrected. This was done for the following authors selected for our Top 100 Authors list: Hansjochem Autrum, Robert R. Capranica, François Clarac, Joachim Erber, Walter Heiligenberg, Franz Huber, Nicholas Justin Marshall, Jürgen J. Milde, Robert M. Olberg, Heiner Römer, Klaus Schildberger, Andrea Megela Simmons, Karl von Frisch, and Hong Young Yan.

The articles of some authors appeared in both JCPA and JCPB. Among the individuals selected for the Top 100 Authors list, this is the case only for Stephan Steinlechner. He published two papers in JCPB and four papers in JCPA. Since the majority of his articles appeared in JCPA, we have analyzed all his papers, independent of the sister journal, as publications in JCPA.

Another potential source of inaccuracies in the author and citation data may stem from errors of the information extracted from scanned (older) articles for which no digital metadata are available.

For each of these performance criteria, authors were ranked in descending order (Table 1, 2, 3). In the first step, the name of the top author in each of these three lists (Friedrich G. Barth, Randolf Menzel, and Colin Stephenson Pittendrigh) was selected. In the second step, after selection of Doekele G. Stavenga from the top-prolific-authors’ list, Serge Daan as the next-ranked author in the total-number-of-citations list was chosen because Colin Stephenson Pittendrigh had been already selected in the previous step. Likewise, from the mean-citation-rate list, Horst Hertel was chosen instead of Serge Daan. The equivalent procedure was repeated until the 54th iteration step. By then, the top 99 authors had been identified. In the 55th iteration step, the authors available for selection from the three performance-criteria lists were Masakazu Takahata, Henning Scheich, and Carl D. Hopkins. Among them, the top-ranked author was selected based on all three performance criteria by awarding 1, 2, or 3 points according to relative rank in each of the three categories. The author with the largest number of points across these three categories, and thus the 100th individual on our Top 100 Authors list, was Henning Scheich.

**Table 1:** The top prolific authors of the *Journal of Comparative Physiology A* and its predecessors over the last 100 years

Rank	Name	Total number of publications
1	Friedrich G. Barth	64
2	Doekele G. Stavenga	46
4	Rüdiger Wehner	42
4	Bernhard Ronacher	42
4	Horst Bleckmann	42
6.5	Randolf Menzel	41
6.5	Jörg-Peter Ewert	41
8	Walter F. Heiligenberg	40
9	Heiner Römer	36
10	Jeffrey M. Camhi	35
11.5	Thomas Stephen Collett	32
11.5	Peter M. Narins	32
13	Hansjochem Autrum	32
14.5	Kurt Hamdorf	31
14.5	Günther K.H. Zupanc	31
16	Hans-Ulrich Schnitzler	30
17	Manfred Kössl	28
19.5	Mandyam Veerambudi Srinivasan	26
19.5	Albert S. Feng	26
19.5	Thomas W. Cronin	26
19.5	Kentaro Arikawa	26
22	Michael Francis Land	25
23.5	Philip Hung-Sun Jen	24
23.5	Eric James Warrant	24
25.5	Jochen Zeil	23
25.5	François Clarac	23
28.5	John G. Hildebrand	22
28.5	Wolfgang Otto Friesen	22
28.5	Michael Menaker	22
28.5	Joachim Erber	22
34	Joseph A. Bastian	21
34	Theodore Holmes Bullock	21
34	Hanna Mustaparta	21
34	Werner Nachtigall	21
34	David C. Sandeman	21
34	Andrew Stanton French	21
34	Toshiki Nagayama	21
41.5	Martin Lindauer	20
41.5	Ronald R. Hoy	20
41.5	Nicholas Mrosovsky	20
41.5	Howard Carl Gerhardt	20
41.5	Roy E. Ritzmann	20
41.5	George Adrian Horridge	20
41.5	Robert R. Capranica	20
41.5	Andrea Megela Simmons	20
50	Franz Huber	19
50	Simon Barry Laughlin	19
50	Wolfgang Wiltschko	19
50	Roswitha Wiltschko	19
50	Almut Kelber	19
50	Marianne Vater	19
50	Uwe Homberg	19
50	Gary J. Rose	19
50	James Howard Fullard	19

Publications reflect any type of article, including Original Papers, Short Communications, Review Articles, Review-History Articles, Perspectives, Obituaries, Book Reviews, and Editorials. Authors with tied number of publications have been assigned the same average rank

**Table 2:** The top authors of the *Journal of Comparative Physiology A* and its predecessors over the last 100 years, ranked according to the total number of citations attracted by their papers published in the Journal

Rank	Name	Total number of citations
1	Randolf Menzel	4821
2	Colin Stephenson Pittendrigh	3717
3	Serge Daan	3714
4	Thomas Stephen Collett	3589
5	Rüdiger Wehner	3375
6	Jörg-Peter Ewert	3016
7	Michael Francis Land	2572
8	Walter F. Heiligenberg	2244
9	Friedrich G. Barth	1932
10	Misha Mikhail Vorobyev	1856
11	Hans-Ulrich Schnitzler	1847
12	Lars Chittka	1758
13	John G. Hildebrand	1753
14	Martin Giurfa	1749
15	Mandyam Veerambudi Srinivasan	1678
16	Franz Huber	1657
17	Martin Lindauer	1568
18	Doekele G. Stavenga	1528
19	Simon Barry Laughlin	1482
20	Horst Bleckmann	1449
21	Joseph A. Bastian	1426
22	Wolfgang Wiltschko	1387
23	Gene E. Robinson	1383
24	Hansjochem Autrum	1362
25	Roger Clayton Hardie	1359
26	Heiner Römer	1351
27	Martin Heisenberg	1322
28	Jeffrey M. Camhi	1310
29	Jochen Zeil	1301
30	Robert R. Capranica	1296
31	Michael Menaker	1293
32	Ronald R. Hoy	1194
33	Nicholas Justin Marshall	1181
34	Nicholas Mrosovsky	1178
35	Howard Carl Gerhardt	1152
36	Julian C. Partridge	1151
37	Roswitha Wiltschko	1146
38	Nicholas James Strausfeld	1111
39	Roy E. Ritzmann	1101
40.5	Nathan Scott Hart	1083
40.5	Joachim Erber	1083
42	Bernhard Ronacher	1081
43	Albert S. Feng	1066
44	Theodore Holmes Bullock	1065
45	Thomas A. Christensen	1049
46	Peter M. Narins	1040
47	Thomas W. Cronin	1020
48	Klaus Lunau	1008
49	Harold Leslie Atwood	1006
50	Horst Hertel	1003
51	Douglas M. Neil	1002
52	Günter Ehret	998
53	Hanna Mustaparta	996
54	William J. Davis	989
55	Henning Scheich	975

Authors with tied number of publications have been assigned the same average rank

**Table 3:** The top authors of the *Journal of Comparative Physiology A* and its predecessors over the last 100 years, ranked according to the mean citation rate of their papers published in the Journal

Rank	Name	Mean citation rate
1	Colin Stephenson Pittendrigh	531.0
2	Serge Daan	371.4
3	Horst Hertel	167.2
4	Lars Chittka	159.8
5	Misha Mikhail Vorobyev	154.7
6	Roland Hengstenberg	146.5
7	Nathan Scott Hart	135.4
8	John Manuel De Souza	134.2
9	Reinhard Blickhan	124.7
10	Randolf Menzel	117.6
11	Martin Giurfa	116.6
12	Thomas Stephen Collett	112.2
13	Martin Lindauer	111.8
14	Martin Heisenberg	110.2
15	Russell Grant Foster	108.8
16	Gunther S. Stent	107.7
17	Julian C. Partridge	104.6
18	Michael Francis Land	102.9
19	Gerbera Nalbach	102.3
20	Karl von Frisch	97.3
21	James Keith Bowmaker	96.1
22	Thomas A. Christensen	95.4
23	Nicholas James Strausfeld	92.6
24	Reinhard Wolf	92.3
25	Gene E. Robinson	92.2
26	Nicholas Justin Marshall	90.9
27	Daniel Colaço Osorio	88.4
28	Franz Huber	87.2
29	Heinz Breer	86.5
30	Stephan Steinlechner	83.8
31	Douglas M. Neil	83.5
32	Hong Young Yan	83.3
33	Brian Mulloney	80.8
34.5	Gerald Langner	80.5
34.5	Klaus Schildberger	80.5
36	Rüdiger Wehner	80.4
37	Roger Clayton Hardie	79.9
38	John G. Hildebrand	79.7
39	Robert M. Olberg	79.5
40	Simon Barry Laughlin	78.0
41	Klaus Lunau	77.5
42	Harold Leslie Atwood	77.4
43	Günter Ehret	76.8
44	Allen Israel Selverston	76.7
45	Jürgen J. Milde	76.1
46	William T. Keeton	74.0
47	Jörg-Peter Ewert	73.6
48	Edmund A. Arbas	73.1
49	Wolfgang Wiltschko	73.0
50	Melvin L. Kreithen	73.0
52	Allan Whitenack Snyder	71.8
52	Dora Fix Ventura	71.8
52	Wulfila Gronenberg	71.8
54	Olav Sand	71.0

Authors with tied citation rates have been assigned the same average rank

Nine of the Top 100 Authors are represented on each of the three criteria lists, while 44 authors are included on two lists, and 47 authors appear on one list. The authors that met each of the three selection criteria for inclusion in the Top 100 Authors list are Thomas Stephen Collett, Jörg-Peter Ewert, John G. Hildebrand, Franz Huber, Michael Francis Land, Simon Barry Laughlin, Martin Lindauer, Randolf Menzel, and Rüdiger Wehner.

## The Top 100 Authors list possesses not only notable but also disturbing features

The list of the Top 100 Authors reveals three notable features: First, absence of virtually any author who was scientifically active during the first 50 years of JCPA’s existence; second, exclusive presence of authors who were born at least five decades ago; and third, severe gender imbalance.

### Investigators who were research active during the first 50 years of JCPA’s existence are (almost) entirely absent from the Top 100 authors list

The vast majority of authors on the Top 100 list published in JCPA primarily during the second 50 years of its existence, i.e., between 1974 and 2023. The only exceptions are Karl von Frisch, who published all his papers in the first half of JCPA’s 100 years of history; Hansjochem Autrum, whose last original research paper appeared in 1974; and Kurt Hamdorf, who published of his 31 articles in JCPA 18 between 1960 and 1973, and 13 between 1976 and 1992.

The phenomenon that 97% of the Top 100 Authors published exclusively, or primarily, during the second half of the 100 years of JCPA’s history can be explained by two peculiarities that distinguish the ‘early’ investigators from the ‘modern’ investigators. First, the number of papers of the early investigators was much lower than the number of papers produced by scientists of comparable peer recognition during the last 50 years. This discrepancy, in turn, has resulted in a lower number of citations. The deficit in the number of articles published in JCPA is not compensated by the much longer time that the early publications have been available for attracting citations because papers typically are cited most frequently during the first few years after their publication. Even Karl von Frisch, who was considered a highly prolific author of journal articles and books during his lifetime, published just 9 papers in JCPA. He made it onto the Top 100 Authors list based on the mean citation rate only.

A second feature that sets apart the early investigators from the modern ones is that they very rarely produced papers with more than two authors, and on most of their papers they appeared as single authors (for a detailed analysis of this phenomenon see Zupanc 2015). Since we scored authorship independently of the number of co-authors, our listing gives preference to individuals who have co-authored (as opposed to single-authored) articles. This bias is particularly extreme if the number of co-authors is large and the average contribution of a co-author to these papers is small, compared to the investments that a single author must make to conduct the entire research and write the paper.

### It takes (almost) a lifetime to make it on the Top 100 Authors list

Besides the absence of early investigators (with the exception of Karl von Frisch) on our list, a notable feature characteristic of each of the Top 100 Authors is that they were born at least five decades ago. Of those authors who are still alive, and we know the year of birth, Nathan Hart, born in 1973, is the youngest. In most cases, this reflects the significant amount of time it takes to produce enough papers and/or attract enough citations to be included in the Top 100 Authors list. Only four of the authors who were selected based on the total number of citations have published 10 or less papers in JCPA. They include Colin Stephenson Pittendrigh and Serge Daan who published 7 and 10 papers, respectively. Nevertheless, these two authors top the mean citation rate list with 531 and 371, respectively. Five of their papers, each jointly authored, appeared in a single issue of JCPA, in October1976 (Pittendrigh and Daan 1976a, b, c; Daan and Pittendrigh 1976a, b). Reflecting the enormous impact that these five articles have had, this issue is frequently referred to as the ‘Bible of Chronobiology.’

### Women scientists are disturbingly underrepresented on the Top 100 Authors list

Among the Top 100 Authors, there are only 7 women (Almut Kelber, Hanna Mustaparta, Gerbera Nalbach, Andrea Megela Simmons, Marianne Vater, Dora Fix Ventura, Roswitha Wiltschko). This finding comes as no surprise, given the severe underrepresentation of women among biology faculty during the last 100 years. We hypothesize that this disturbingly low number of women on our list primarily reflects the lack of adequate opportunities that women have traditionally encountered for a long time as they tried to establish their own laboratories and research programs. Without this foundation, they were deprived of the essential instrument for making it to the Top 100 Author list—the production of a large number of papers that attract a large number of citations. Two women scientists who had the potential to become leading zoologists of their times were Ruth Beutler and Ingeborg Beling. Both are portrayed in this Special Issue (Zupanc 2023; Beer et al. 2024). Beutler was never considered for appointment to a faculty position that would have enabled her to establish her own laboratory. For many years, she was employed by Karl von Frisch only as a technician at his institute—after she had received her PhD and even the *Habilitation*. Beling made a breakthrough discovery—time memory in honeybees—during her thesis research and established an excellent publication record after she received her PhD under von Frisch’s mentorship. However, she left science when she married, presumably due to political and/or societal pressure that even forced highly qualified women (like her) into the role of housewives.

In our metanalysis, we also considered the possibility that even women who succeeded in establishing their own publication records were still disadvantaged by gender bias in citation practice. This issue was recently highlighted by Dworkin et al. (2020) who analyzed the papers of five neuroscience journals (*Brain*, *Journal of Neuroscience*, *Nature Neuroscience*, *NeuroImage*, and *Neuron*). They found that reference lists tended to include more papers with men as first and last authors than was expected if gender were unrelated to referencing. Prompted by this finding, we first compared the mean citation rates of the articles of the five women who qualified for inclusion in the Top 100 Authors list by number of publications in JCPA (i.e., independent of any citation record) with the mean citation rates of all 54 authors in this criterion list. The median values of these two groups were almost identical (41.8 for all 54 authors vs. 40.9 for the five women). The publications of two of these five women exhibited mean citation rates above the overall median, whereas the mean citation rates of the publication of the other three women were below the overall median. Then, we took a closer look at the mean citation rates of the papers in JCPA. Two of the seven women had been selected based on this criterion. The value of this performance indicator of one woman was higher (102.3) than the median of the mean citation rate of all 54 authors in this selection category (87.8), while the corresponding value of the other woman author was lower (71.8). Thus, while we would like to stress the limitation of our analysis due to the small sample size, we have not found an indication of pervasive gender imbalance in citation practice toward papers published in JCPA by women, compared to men.

We believe that the latter finding, together with the significant increase in the number of women principal investigators in neuroethology and related disciplines over the last few decades, gives reason for hope. If a list of top authors would be compiled, let’s say at the 125th anniversary of JCPA, the gender distribution among these individuals is likely to be more balanced than it is today.

### The Top 100 Authors: their academic careers and major scientific achievements

In the following, we list the Top 100 Authors in alphabetical order. For each of them, we present biosketches that highlight milestones of their academic careers and scientific achievements. The main sources of the biographical information were autobiographies and obituaries, the authors themselves and/or collaborators, as well as public information from the Internet. In some instances, the amount of information available was rather limited, which has unfortunately resulted in imbalances in length of the biosketches. These differences should not be interpreted as differences in the significance of the contributions made by authors to scientific knowledge.

To indicate links between different authors, we have marked names of authors mentioned in biosketches other than their own with an asterisk.

**Edmund A. Arbas** (1950–95) earned his PhD from the University of Oregon in 1980 studying the muscular and neural correlates of flight loss in a grasshopper, supervised by Graham Hoyle. His interest in pattern-generating networks led him to pursue postdoctoral training studying the neural mechanisms of heartbeat and its modulation in the leech with Ronald Calabrese at Harvard University. Following a second postdoctoral position with Barry Ache at the University of Florida, where he performed intracellular recordings from olfactory interneurons in the crayfish, he became assistant (1986) and later associate professor at the Arizona Research Laboratories, Division of Neurobiology at the University of Arizona. Here he continued research on muscle degeneration in grasshoppers but also became interested in insect olfactory coding. Five of his 10 papers in JCPA contributed significantly to research on moth olfactory behavior and central nervous coding of pheromone signals in the moth brain. In 1995, while on a sailing trip with his family in Mexico, he died in a tragic accident.

**Kentaro Arikawa** (1957-) was born in Tokyo. He is professor and executive director of the Research Center for Integrative Evolutionary Science at Sokendai, Japan. Arikawa studied natural science at Jiyu Gakuen College and received his PhD in behavioral biology (1986) from Sophia University, analyzing the unique photoreceptive system of butterfly genitalia. He continued to work on vision in butterflies as research assistant and later full professor at Yokohama City University, interrupted by research positions at Indiana University and the Australian National University in Canberra. In 2006, he moved to his current position at Sokendai. Arikawa has received several prestigious awards, including the National Medal with Purple Ribbon from the Emperor of Japan in 2022. His continuing interest (as he puts it himself) is “*how do animals see the world”*. His studies on color vision in insects, mostly in *Papilio* butterflies, are comprehensive and include anatomical, ultrastructural, physiological and behavioral techniques. He has served on the editorial board of JCPA since 2009 and has contributed 26 highly cited papers on the visual behavior and photoreceptor physiology of butterflies.

**Harold L. Atwood** (1937-) was born in Montreal. His academic training was in biology and neurophysiology, and he studied at the University of Toronto (BA, 1959), the University of California Berkeley (MSc, 1960), and the University of Glasgow (PhD, 1963; DSc, 1979), where he worked with C.M. Yonge and Graham Hoyle. He conducted postdoctoral research with Graham Hoyle at the University of Oregon and with C.A.G. Wiersma at the California Institute of Technology. Atwood joined the faculty of the University of Toronto in 1965, and remained there until his retirement in 2002. He directed the Medical Research Council of Canada Research Group in Nerve Cells and Synapses. His many awards include a Guggenheim Fellowship (1972), Fellow of the American Association for the Advancement of Science (1981), Fellow of the Royal Society of Canada (1982), Distinguished Scientist of the Medical Research Council of Canada (1997), and Fry Medal of the Canadian Society of Zoologists (1997). Atwood’s 13 publications in JCPA concern the physiology of muscular contraction, motor control, synaptic transmission, and synaptic modification (long-term facilitation and low-frequency depression) in the nervous and neuromuscular systems of crustaceans and fruit flies.

**Hansjochem Autrum** (1907–2003) was a giant in the field of sensory physiology for most of the twentieth century. He was the editor-in-chief of JCPA from 1960–96 (he was also editor-in-chief of *Naturwissenschaften* in parallel). He studied mathematics and physics (with a minor in biology) at the Humboldt University in Berlin, and received a PhD under the supervision of Richard Hesse in 1931. After the Second World War, Autrum became adjunct professor at the University of Göttingen (1948), full professor at the University of Würzburg (1952), and Head of the Zoological Institute of the Ludwig Maximilian University of Munich (LMU Munich) (1958). He was also highly active in science politics and was instrumental in the foundation of three major German universities: University of Regensburg, University of Bayreuth, and University of Konstanz. He was elected as a member of the German National Academy of Science (Leopoldina) in 1957 and as a member of the Bavarian Academy of Sciences in 1958. He received the Feldberg Foundation Prize in 1966, was admitted to the order Pour le Mérit for Sciences and the Arts in 1977, and received the Bavarian Maximillian Order for Science and Art in 1984. Autrum made groundbreaking foundational studies on the sensory systems of animals, including the discovery of the extraordinarily low vibrational sensitivity thresholds of bush crickets and cockroaches (with threshold amplitudes in the order of atomic diameters!). He also made seminal studies of the physiological properties of insect eyes, including their temporal and spectral sensitivities, and was a pioneer of intracellular recordings in photoreceptors.

**Friedrich G. Barth** (1940-) was born in Munich. He attended LMU Munich for his undergraduate studies in zoology, botany, physics, chemistry, and human physiology from 1959–62. Subsequently, he went to the University of California Los Angeles, to join the laboratory of Theodore H. Bullock* as a Fulbright Scholar. After returning to LMU Munich, he obtained his PhD under the mentorship of Hansjochem Autrum* in 1967, followed by the *Habilitation* in 1971. Between 1974 and 1987, he served on the faculty of Goethe University Frankfurt, and from 1987–2008 he was professor and chair of the Department of Neurobiology at the University of Vienna. Since 2008, he has been emeritus professor. During his scientific career, Barth has addressed important biological questions, particularly in the context of social communication, by combining sensory physiology with physics and engineering; and by complementing laboratory experiments with field observations in search of matches between properties of sensors and behavior and ecology. One of his major research interests has been vibratory communication in spiders and the functional biomechanics of cuticular strain receptors (a class of sensors that includes the spider vibration receptor). Since the early 2000s, he has increasingly turned his attention to the analysis of the signals and cues used by stingless bees for recruitment of nestmates to food sources—research that was largely based on fieldwork in Brazil. Barth has been closely affiliated with JCPA as both author and editor. His first paper, a physiological characterization of a phasic-tonic proprioceptor in the telson of the crayfish, appeared 60 years ago (Barth 1964). His last papers, including the most frequently accessed article of the last 100 years (Dyer et al. 2021), were published in 2021. In total, Barth has authored 64 publications in JCPA, which distinguishes him as the most prolific JCPA author of all times. Perhaps even more impactful was his service as editor-in-chief, a responsibility he assumed in 1996 when Hansjochem Autrum* retired from this position. Barth carried out this task with great dedication over the following 25 years, during which he left his editorial mark on numerous articles published in JCPA.

**Joseph A. Bastian** (1944-) was born in Mare Island, California. After completing his undergraduate education at Elmhurst College (1966), he went to the University of Notre Dame, where he studied, both as a graduate student and as a postdoctoral fellow, bee communication and flight muscle physiology in the laboratory of Harald Esch. Each of the four papers that resulted from this work was published in JCPA. In 1969, he joined the lab of Shigehiru Nakajima at Purdue University to continue postdoctoral training in muscle physiology but, at the same time, he changed organisms from invertebrates to vertebrates. During his third postdoctoral stint, from 1972–74, in the laboratory of Theodore H. Bullock* at the University of California San Diego, Bastian began to address the question that became central to the research program he subsequently established as a faculty member at the University of Oklahoma: how does the central nervous system process electrosensory information in weakly electric fish? In recognition of his accomplishments, he received several distinctions, including the Jacob Javits Neuroscience Investigator Award. He was also named the George Lynn Cross Research Professor by the University of Oklahoma. Currently, Bastian is emeritus professor. The impact of the papers that he published in JCPA is not only evident from their large number (21), but also the many citations they have attracted—over 1400 in total and nearly 70 on average.

**Horst Bleckmann** (1948-) was born in Rietberg, Germany. After successful completion of an apprenticeship as a machinist, he earned the *Abitur* (the German high school diploma) through second-chance education. He then studied biology and chemistry at the University of Giessen, where he also received his PhD in 1979. Following his *Habilitation* in 1986, Bleckmann worked with Theodore H. Bullock* as a Heisenberg Fellow at the Scripps Institution of Oceanography of the University of California San Diego, and at the University of Bielefeld. In 1994, he was appointed to a full professorship at the University of Bonn, where he served on the faculty until 2017. His research covered a wide range of topics within neurophysiology and sensory physiology, neuroethology, and bionics. However, he is best known for his studies exploring how sensory information perceived through the lateral line system of cartilaginous and bony fishes is processed by the central nervous system. Part of this research led to the development of bionic sensors. Bleckmann has been a prolific author, with 42 highly cited articles published in JCPA. He has also contributed an autobiographical essay to this Special Issue.

**Reinhard Blickhan** (1951-) was born in Eppertshausen, Germany. He studied physics at the University of Giessen and the Technical University Darmstadt and received his *Diplom* (master’s) degree in 1976. Over the following 40 years, he applied his training in physics to study phenomena in biomechanics. The foundation for this interdisciplinary career was laid during his PhD thesis research on ‘Strain in the exoskeleton of spiders’ in the laboratory of Friedrich G. Barth* at Goethe University Frankfurt, for which he was awarded a *Dr. rer. nat.* degree in 1983. Following postdoctoral training at Harvard University and the *Habilitation* at the University of Saarland, he was appointed professor of biomechanics at the University of Jena in 1993, where he served on the faculty until his retirement in 2016. By combining experimental studies with numerical simulation, a major focus of his research was on the biomechanics of terrestrial and aquatic locomotion. The impact of Blickhan’s work is evident, among other indicators, by the nearly 125 citations that each of his 6 papers published in JCPA have attracted on average.

**James K. Bowmaker** (1946-) is emeritus professor of ophthalmology at University College London (UCL). He was educated at Queen Mary University of London, receiving a bachelor’s degree (1967) and a PhD (1970) in zoology for his work on visual transduction. He then pursued postdoctoral work with Frederick Crescitelli at the University of California Los Angeles (1970–72) and with Herbert Dartnall at the Medical Research Council Vision Unit at the University of Sussex (1972). It was with Dartnall that he first began using microspectrophotometry to measure the absorption characteristics of visual pigments in humans and monkeys. Bowmaker took a faculty position first as lecturer (1977) and then as reader (1987) at Queen Mary College. He moved to the Institute of Ophthalmology at UCL in 1989, becoming professor of vision research in 1994. He was awarded the Rank Prize for optoelectronics (1988) and is an honorary member of the Colour Group (Great Britain). Bowmaker’s research interests focus on the evolution and functions of visual pigments and color vision in vertebrates. In eight papers in JCPA, he presented new data on photoreceptors and visual pigments in several species of birds, fishes, and chameleons. He found that birds and freshwater fish have four visual pigments, including an ultraviolet-sensitive pigment, thereby possessing tetrachromatic color vision.

**Heinz Breer** (1946-) was born in Wieste, Germany. He studied biology and chemistry at the University of Münster from 1968–72 and received his PhD from the University of Hohenheim in 1974. After postdoctoral training at the Max Planck Institute for Biophysical Chemistry in Göttingen, he worked for 10 years as a scientific assistant at the University of Osnabrück, where he received the *Habilitation* in zoology in 1982. In 1987, he was appointed to a professorship in physiology at the University of Hohenheim. During the following three decades of his tenure, Breer also served as vice president of the University and dean of the College of Science. After his retirement in 2018, he was re-appointed to the University as its first senior professor. Breer’s research activities are mainly in neurochemistry, with an emphasis on olfaction and gustation. He published approximately 300 papers, of which six appeared in JCPA. On average, each of the latter publications has been cited nearly 90 times, underscoring the impact of his research.

**Theodore H. Bullock** (1915–2005; for obituaries see Zupanc 2006; Zupanc and Zupanc 2008) was born to American Presbyterian missionaries in Nanking, China. His immersion in the Chinese culture over the first 13 years of his life laid the foundation for his lifelong cosmopolitan outlook. He received his undergraduate degree (1936) and then his PhD in zoology from the University of California Berkeley (1940), working with S.F. Light. He then spent four years at Yale University, with summers at the Marine Biology Laboratory at Woods Hole. There, the ample opportunity to work on a large variety of organisms had a major impact on the development of his interest in comparative physiology. Later, he would argue that examination of the different mechanisms used by various organisms is as important as the search for commonalities in understanding how the brain works. As a consequence, during his tenure as a faculty member at the University of California Los Angeles (1946–66), and the University of California San Diego (1966–2005), Bullock studied an enormous variety of taxa, including cnidarians, crustaceans, fish, amphibians, and reptiles. He was a member of the US National Academy of Sciences (elected 1963), the American Academy of Arts and Sciences (1961), and the American Philosophical Society (1970). He served as the first president of the International Society for Neuroethology (1984), the third president of the Society for Neuroscience (1973–74), president of the American Society of Zoologists (1965) and of the American Association of University Professors (1955–56). He was awarded the Ralph W. Gerard Prize from the Society for Neuroscience (1984) and the Karl Spencer Lashley Award from the American Philosophical Society (1968). Bullock’s research interests were diverse and included, among others, the study of physiological processes at chemical and electrical synapses, the anatomy and physiology of sense organs, the analysis of electrical brain activity associated with cognitive events, and the mathematical modelling of neurophysiological activity. His many ‘firsts’ include the discovery of two new senses in animals—the facial pits of pit vipers that act as thermal sensors, and electroreceptors in the skin of certain fish. Equally important, he inspired numerous students and co-workers all over the world to identify new research directions and to establish their own field of study—an influence that can be felt even today. His 21 articles published in JCPA have attracted nearly 1100 citations.

**Jeffrey M. Camhi** (1941-), born in New York, is professor emeritus of cell and developmental biology and founding director of ‘Nature Park and Galleries,’ an open-campus museum, at the Hebrew University of Jerusalem. Camhi earned his bachelor’s degree at Tufts University and his PhD (1967) at Harvard University under the direction of Ian Cooke. His dissertation examined the operation of wind receptors in the locust. He joined the faculty in the Department of Neurobiology and Behavior at Cornell University in 1967, where he trained Roy Ritzmann*. In 1982, Camhi accepted a faculty position in the Department of Cell and Animal Biology at Hebrew University Jerusalem, where he spent the rest of his academic career. His 1984 book, *Neuroethology: Nerve Cells and the Natural Behavior of Animals* (Sinauer) inspired many students to enter the field. Currently, he is actively involved in developing strategies to improve public outreach and education in science and nature. Camhi’s 35 papers in JCPA contribute a neuroethological analysis of the escape system of the cockroach. He determined the threshold wind velocity needed to evoke the escape response, how these responses are coded by in the nervous system, and developed a model of the escape system based on synaptic responses of different populations of interneurons.

**Robert R. Capranica** (1931–2012) was born in Southern California and was a resident of Tucson, Arizona at the time of his death (see obituary by Adler et al. 2013). After service in the US Navy (1951–54), he received his BA in electrical engineering from the University of California Berkeley in 1958. Capranica then moved to New York University, where he received his MS, and then to the Massachusetts Institute of Technology (MIT) from which he received his ScD in electrical engineering in 1965. At MIT, he studied with Moise Goldstein, Lawrence Frishkopf, and Jerome Lettvin. It was Lettvin’s pioneering work on “what the frog’s eye tells the frog’s brain” that inspired Capranica to examine “what the frog’s ear tells the frog’s brain.” Capranica’s dissertation, published as a monograph titled *The Evoked Vocal Response of the Bullfrog* (MIT Press, 1965), remains a model of how to incorporate engineering and biology to answer questions about evolution and adaptation to the environment. His first position after MIT was at the Bell Telephone Laboratories in Murray Hill, New Jersey (1958–69). He was recruited to Cornell University in 1969, with appointments in the Department of Neurobiology and Behavior and the Department of Electrical Engineering. At Cornell he built a large laboratory dedicated to the integrative study of the auditory system of anuran amphibians. Six of his trainees and collaborators are included among the Top 100 Authors listed here (Günther Ehret*, Albert Feng*, Howard Carl Gerhardt*, Peter Narins*, Gary Rose*, Andrea Megela Simmons*). Capranica served as an associate editor of JCPA from 1974–86, and is one of the founding members of the International Society for Neuroethology. He was an elected Fellow of the Acoustical Society of America (1974) and of the International Society for Neuroethology (2012). He and his spouse Patricia endowed the Capranica Fund at the International Society for Neuroethology to celebrate the achievements of young neuroethologists. Capranica retired from Cornell in 2012 and moved to Arizona. His 20 papers in JCPA integrated field studies and laboratory electrophysiological methods to decipher how species-specific advertisement calls are produced, recognized, and encoded in the brain. These papers quantify the frequency tuning of the frog’s inner ear and how it relates to the spectral content of the advertisement call; sex differences in the tuning of the inner ear; acoustic sensitivity of the frog’s midbrain and thalamus; mechanisms by which small frogs can localize sound sources; and the use of spectroscopy to measure the mechanics of the middle ear.

**Lars Chittka** (1963-, born in Bad Homburg, Germany) is professor of sensory and behavioral ecology at Queen Mary University of London. He was educated at the University of Göttingen and the Free University of Berlin, where he received his PhD under the mentorship of Randolf Menzel*. He is a member of the German National Academy of Science (Leopoldina), and Fellow of the Linnean Society, the Royal Society of Biology, and the Royal Entomological Society. In 2006, he received the latter’s Lesley Goodman Award. Chittka has made seminal contributions to our understanding of insect color vision and the interactions of insects with flowers, and his work on the cognitive abilities of bumblebees has changed the way we understand the evolution of animal cognition and its neural underpinnings. He is well known for his work exploring the behavior, cognition, and ecology of bees, a topic he summarized in his 2022 book *The Mind of a Bee* (Princeton University Press). He has collaborated in several musical and artistic compositions based on bee biology. Chittka’s 11 papers published in JCPA—nearly all of which deal with color vision in bees—have been highly cited. A good example is his 1992 analysis (with Randolf Menzel*) of 180 floral spectral reflection spectra showing that flower colors are well matched to the color vision of bees (Chittka and Menzel 1992, with 304 citations as of November 2023).

**Thomas A. Christensen** (1956-) was born in Queens, New York. He earned his BSc in biology from the State University of New York at Stony Brook and his PhD from the same institution in 1983 for research supervised by Albert Carlson on the neural control of luminescence in the firefly. For postdoctoral training, he joined the group of John Hildebrand* at Columbia University, and moved with him to the University of Arizona in late 1985 to become senior research scientist at the Arizona Research Laboratories, Division of Neurobiology. There, he performed groundbreaking work in analyzing the mechanisms of olfactory signal processing in the brain of the sphinx moths and, in collaboration with Hanna Mustaparta*, characterized the olfactory system of heliothine moths. Motivated by an increasing interest in human studies, in 2006 Christensen moved within the University of Arizona to the Department of Speech, Language and Hearing Sciences to perform MRI studies on attention and language representation in the human brain. In 2012, he left the University of Arizona to take on a teaching position at Pima Community College. In 11 highly cited articles in JCPA, he contributed significantly to our understanding of how pheromone signals are processed in the brain of sphinx moths and heliothine moths.

**François Clarac** is emeritus research director at the French National Center for Scientific Research (CNRS) Aix-Marseille. He continues to teach at Aix-Marseille and at Montpellier. He is a Member of the Academy of Europe (elected 1996) and the Académie de Marseille (elected 2006). He served as Chancellor of the Académie de Marseille in 2011, and as Director in 2012 and 2014. Clarac’s research focuses around cellular mechanisms of locomotion, neural bases of rhythmic behavior, and developmental plasticity of the spinal cord. He has collaborated with Douglas M. Neil* on comparative studies of the swimmeret system in crustaceans. Currently, Clarac continues to write and speak on the history of neuroscience and on the importance of neuroscience research. Together with Jean-Peirre Ternaux, he has authored two books, *Encyclopédie historique des neurosciences: Du neurone à l'émergence de la pensée (Historical Encyclopedia of Neuroscience: From Neuron to the Emergence of Thought;* de boeck, 2008) and *Le Bestiaire cérébral (The Cerebral Bestiary, CNRS Editions,* 2012).

**Thomas S. Collett** (born 1939 in London) is emeritus professor of neurobiology at the University of Sussex. He studied psychology and zoology at University College London, and received his PhD with David Blest working on visual behavior of moths. Inspired by Hubel and Wiesel’s work on the visual cortex, they pioneered physiological recordings from motion-sensitive interneurons in moths. In 1965, Collett was appointed assistant lecturer at the University of Sussex and later progressed to the rank of full professor. He is a Fellow of the Grass Foundation (1965). Collett has contributed significantly to the study of insect vision, spatial orientation, and navigation. His interests focus on behavioral studies of navigation, landmark learning, and spatial memory in flies, ants, and bees. His meticulous observations on landmark learning in bees and ants, as well as his studies on visual control of flight behavior in hoverflies, received record numbers of citations. Out of well over 170 papers, he published 32 highly cited articles on these topics in JCPA.

**Thomas Cronin** (1945-) is renowned for his decades of research on the visual systems of crustaceans, and particularly on the remarkable polarization and color vision of mantis shrimps. Educated at Dickinson College (BSc, 1967) and Duke University (MA, 1969; PhD, 1979), he studied crustacean visual pigments in the lab of Timothy Goldsmith at Yale University before joining the University of Maryland Baltimore in 1983, where he is now professor of biological sciences. Cronin’s chief interest is understanding how the visual systems of animals are matched to their habitats and lifestyles, a field known as ‘visual ecology.’ Together with Sönke Johnsen, Justin Marshall*, and Eric Warrant*, Cronin authored a book on this topic—*Visual Ecology* (Princeton University Press, 2014)—which is now the most important work in the field. For his many contributions to visual ecology, Cronin was elected as Fellow of the International Society of Neuroethology in 2014. Cronin’s many well-cited papers in JCPA deal with color and polarization vision in mantis shrimps, mysids, crayfish and insects, with notable contributions to our understanding of how visual pigments are spectrally tuned for vision in specific light environments (e.g., at different depths in the ocean) and for different tasks (e.g., for detecting specific colors of bioluminescence).

**Serge Daan** (1940–2018; see obituary by Trillmich et al. 2018) was an inspiring scientist who contributed seminal work to the fields of chronobiology, sleep research, psychiatry, physiology, ecology, and behavioral biology. Daan studied biology at the University of Amsterdam and received his PhD on hibernation of bats and mice in 1973. In 1971, while still finishing his PhD thesis, he became a postdoctoral researcher with Jürgen Aschoff at the Max Planck Institute for Behavioral Physiology in Erling-Andechs. The time with Aschoff founded his lifelong interest in the role of circadian clocks in seasonal timing and their significance in an ecological context. Through Aschoff, Daan met Colin Stephenson Pittendrigh* with whom he spent his second postdoctoral position at Stanford University. During his stay at Stanford, he wrote a series of five papers together with Pittendrigh (‘A functional analysis of circadian pacemakers in nocturnal rodents,’ Parts I-V), which appeared as a single issue of JCPA in 1976. This series of papers belongs to the highest cited research papers in JCPA and has become the ‘Bible of Chronobiology’ for hundreds of students and scientists. In 1975, Daan moved to the University of Groningen where he was appointed professor of ethology in 1996. In 2003, he was promoted to the prestigious Niko Tinbergen Chair in Behavioral Biology, which he held until his retirement in 2011. He was awarded several prizes and awards, including the International Prize for Biology (2006), conferred by the Emperor of Japan. He was appointed Knight in the Order of the Dutch Lion (2005) and was elected Foreign Fellow of the Royal Society of Canada (2000). Daan (co-) supervised more than 40 PhD students. Most of them pursued successful careers in science. His work became more eco-physiologically oriented by focusing on kestrel-vole interactions in relation to energy expenditure. ‘Time and Energy’ was a central theme in his Chronobiology group. EEG recordings during sleep made together with Alex Borbély and Domien Beersma led to the development of the ‘Two Process Model’ of human sleep regulation, a model which still inspires many sleep researchers all over the world. He stayed active after retirement. His last publication, the biography of Jürgen Aschoff—*Die Innere Uhr des Menschen: Jürgen Aschoff (1913–1998), Wissenschaftler in einem bewegten Jahrhundert* (*The Biological Clock of Humans: Jürgen Aschoff (1913–1998), Scientist in a Turbulent Century*; Reichert, 2017), appeared two months before his death. He has published 10 highly-cited papers in JCPA.

**William Jackson Davis** is founder and executive director of the Environmental Studies Institute. He received his BA in zoology (1964) at the University of California Berkeley and his PhD in biology (1968) under the direction of Graham Hoyle at the University of Oregon. Davis pursued postdoctoral research with Melvin Cohen (University of Oregon, 1967–68) and with Donald Kennedy (Stanford University, 1968–70), where he investigated motor control in lobsters. He joined the Department of Biology at the University of California Santa Cruz in 1969, and remained there as professor of ecology and evolution and of environmental sciences until 2004. In 1981, Davis founded the non-profit Environmental Studies Institute. The mission of this institute is to research and consult on matters of importance to public policy, such as radioactive waste, nuclear proliferation, climate change, and biodiversity. Davis has published extensively in the areas of biological sciences, environmental sciences, health and fitness, and public policy. His 17 papers in JCPA analyze the hormonal and neural control of motor behavior in invertebrates, with an emphasis on the physiology of command neurons in the buccal ganglion of sea slugs; the role of internal states in controlling slug feeding behavior; and the neural and behavioral control of walking and swimmeret motion in lobsters.

**John Manuel De Souza** (1930–2016) was professor at the Institute of Psychology at the University of São Paolo. He earned undergraduate degrees in civil and electrical engineering at Mackenzie Presbyterian University in Sao Pãolo (1955). After a career in business, in 1983 he returned to academia, enrolling in the University of São Paolo. He received his master's (1986) and PhD (1993) degrees in experimental psychology, working with Dora Fix Ventura* on the neural coding of visual cues in the retina and first visual ganglion of Hymenoptera. He continued to collaborate with Ventura throughout his career on projects related to the spectral sensitivity and morphology of insect compound eyes and the electrophysiology of the visual system. In 1984 and 1985, de Souza worked with Randolf Menzel* at the Free University of Berlin to characterize and compare color vision sensitivity of photoreceptors in closely related hymenopterans. His six publications in JCPA describe the use of intracellular recordings to elucidate the operation of photoreceptor cells and other retinal cells in multiple hymenopteran species; how the color vision system of these animals has evolved to adapt to different ecologies and behavioral demands; and to characterize the ultraviolet sensitivity of the goldfish retina.

**Günter Ehret** (born 1949, Kaiserslautern, Germany) is professor emeritus for neurobiology at the University of Ulm, Germany. Ehret studied chemistry and biology at the Technical University of Darmstadt and received his doctorate (*Dr. rer. nat.*) in 1975 for studies on the auditory system of mice, supervised by Hubert Markl. He moved as research assistant with Markl to the University of Konstanz, where he received his *Habilitation* (1981) followed by a Heisenberg Stipend (1982–85) from the German Science Foundation. As a visiting professor at Cornell University, he conducted behavioral and electrophysiological studies on hearing in frogs in conjunction with Robert R. Capranica*, and at the University of California San Francisco, he examined neural coding of complex sounds in the inferior colliculus of cats in collaboration with Michael Merzenich. In 1993, Ehret received the Merckle Research Award for Natural Science. In 1988, he became professor for neurobiology at the University of Ulm where he stayed until retirement in 2014. Ehret’s scientific interests focus on the auditory system and acoustic communication in mice, but he also contributed to studies in insects and frogs. In more recent years, his interest shifted to evolutionary and emotional aspects of auditory communication and the neural basis of awareness and consciousness. Out of well over 100 publications in peer-reviewed journals, numerous book chapters, and two books, he published 13 papers in JCPA and, together with Henning Scheich*, he edited an extremely well-received Special Issue on *Auditory Cortex* (Volume 181, Issue 6, 1997).

**Joachim Erber** (1946-) was born in Wiesbaden, Germany. He studied electrical engineering at the Technical University of Darmstadt. In his *Diplom* (master’s) thesis he developed a model of photoreceptor function. He subsequently shifted further into the field of zoology and completed his dissertation, supervised by Randolf Menzel* and Hubert Markl, on learning dynamics in the honeybee. As a postdoctoral researcher in Menzel’s group, Erber performed electrophysiological studies on interneurons in the brain of bees and shore crabs, and spent a year at the Australian National University studying the visual interneurons of crayfish. In 1976, together with Menzel, he moved as assistant professor to the Free University of Berlin and continued behavioral and electrophysiological studies on olfactory learning in honeybees and visual signal processing in crabs. Following his *Habilitation* in 1978 and a research stay at the University of Sussex, Erber became professor of animal physiology at the Technical University of Berlin in 1982. There, he developed various learning paradigms for honeybees and studied the underlying neural mechanisms. In his later years, a focus of his research became the involvement of biogenic amines, and the identification of amine receptors, in the honeybee brain. In collaboration with Robert Page at Arizona State University, he analyzed the physiological basis of differences in honeybee foraging behavior. Erber retired in 2011. He published his data on learning assays, the effect of sensory thresholds on learning, and the role of biogenic amines on honeybee learning in 22 articles in JCPA.

**Jörg-Peter Ewert** (1938-) is one of the pioneers of neuroethology who unraveled the neurophysiological bases of visually controlled behavior in the toad. Ewert was born in Danzig and studied biology, chemistry and geography at the University of Göttingen. After graduation (1965), he worked as a scientific assistant at the Zoological Institute of the Technical University of Darmstadt until 1969, first under Wolfgang Luther and then under Hubert Markl. During a research visit at the Free University of Berlin, Ewert learned electrophysiological recordings and began to record from neurons in the visual system of the common toad. He obtained his *Habilitation* from the Technical University of Darmstadt in 1969. From 1970–71, he worked with David J. Ingle at the Harvard Medical School, and from 1971–72 as a university professor at the Zoological Institute of the Technical University of Darmstadt. In 1973, Ewert became the chair of zoology/physiology at the Faculty of Natural Sciences at the University of Kassel, where he formed a neuroethology research team and where he remained until his retirement in 2006. In 1980, he published his book *Neuroethology: An Introduction to the Neurophysiological Fundamentals of Behavior* (Springer, 1980), which inspired many neuroethologists. He is an elected Fellow of the American Association for the Advancement of Science (1983). Ewert’s 41 papers published in JCPA describe, analyze, and model the release of visual behaviors in prey-catching toads.

**Albert S. Feng** (1944–2021; for an obituary see Narins and Feng 2023) made fundamental discoveries in neuroethology and comparative physiology, probing acoustic communication in frogs, electroreception in electric fishes, and echolocation in bats. Feng was born in Bandung, Indonesia, and moved to the US to study electrical engineering at the University of Miami. He earned his BA in 1968 and his master’s in 1970. He received his PhD in electrical engineering (1975) at Cornell University under the mentorship of Robert R. Capranica*. Feng pursued postdoctoral studies on electroreception (1976) with Theodore H. Bullock* at University of California San Diego and on echolocation (1977) with James A. Simmons at Washington University. He joined the faculty at the University of Illinois in 1977, remaining there until his retirement in 2010. At Illinois, he served as department head of molecular and integrative physiology and as director of the neuroscience program at the Beckman Institute. He played an essential role in building and expanding Beckman’s neuroscience program, and was highly respected as a teacher and educator. He traveled extensively to conduct field work on vocalizing frogs and echolocating bats. Feng was an elected Fellow of the Acoustical Society of America (1997), the American Association for the Advancement of Science (1993), and the Alexander von Humboldt Foundation (1988). His 26 papers in JCPA analyze perception and localization of advertisement calls by frogs, neural coding of these advertisement calls, and neural mechanisms of target ranging in echolocating bats. This work inspired his development of a novel hearing aid for canceling out background noise. Along with Peter M. Narins*, Feng was a member of the team that discovered the ability of some specialized frog species to hear ultrasound. A Special Issue devoted to him and his work (*Neuroethology of Auditory Systems: Contributions in Memory of Albert S. Feng*) was published in JCPA (Volume 209, Issue 1, 2023).

**Russell G. Foster** (1959-) is the director of the Nuffield Laboratory of Ophthalmology, the Head of the Sleep and Circadian Neuroscience Institute, and a Nicholas Kurti Senior Fellow at Brasenose College at the University of Oxford. He studied at the University of Bristol, receiving his PhD in 1984 for a thesis on how extraretinal photoreceptors mediate photoperiodic induction in Japanese quail. Furthermore, he showed that the pineal eye of *Xenopus* can directly excite behavior, work that was published in JCPA in 1982. From 1988–95 he conducted postdoctoral work at the University of Virginia with Michael Menaker*. During this time, Foster, Martin Ralph, and Menaker carried out their impactful transplantation experiments that showed that the mammalian suprachiasmatic nucleus is sufficient and necessary for mammalian circadian rhythms. In addition, Foster found that mice with degenerated retinas can still synchronize their circadian rhythms to light–dark cycles, providing evidence that rods and cones are not necessary for circadian entrainment. This seminal paper appeared in JCPA in 1991 and was highly cited. Later he showed that melanopsin in the retinal ganglion cells works as a circadian photoreceptor. In 1995, Foster returned to the UK and started his own laboratory at Imperial College in London where he became chair of molecular neuroscience within the Faculty of Medicine. Later, he transferred his laboratory to the University of Oxford to engage in translational research, investigating the importance of circadian clocks and sleep for human health. He has been awarded many prizes, and his contribution to science has been recognized by his election as Fellow of the Royal Society and as Fellow of the Academy of Medical Sciences, culminating in the 2015 New Year’s honors with the award of Commander of the Order of the British Empire. Foster has published more than 100 research articles, four popular science books and writes newspaper articles. He regularly contributes to radio and television.

**Andrew S. French** (1943-) was born in Holbeton, UK. He studied chemistry at the Universities of Salford and Essex and received a PhD in chemistry from the University of Essex in 1968, supervised by John N. Bradley. For postdoctoral training he joined the laboratory of Richard Stein at the University of Alberta. There, he soon moved through the ranks to full professor of physiology in 1982, interrupted only by a sabbatical at the Australian National University. In 1993, he became professor and head of the Department of Physiology and Biophysics at Dalhousie University, where he is (since 2022) professor emeritus. French’s research covers a wide range of topics related to sensory transduction and signal transmission in sensory systems, in particular the visual system of insects and mechanosensory system of insects and spiders. In his studies he characterizes ion channels involved in signal transduction, the role of neurotransmitters, as well as the information content of sensory channels. From 1989–90, he served as president of the Canadian Physiological Society and in 2009 received an honorary doctorate from the University of Oulu. Of his nearly 200 published journal articles and more than 20 book chapters, 21 papers on phototransduction in cockroaches and flies, and signal transduction in spider and cockroach mechanoreceptors, among other topics, appeared in JCPA.

**W. Otto Friesen** (1942-) was born in Elbing, Germany. After immigrating to the United States, he received his BA from Bethel College in 1964, his MA from the University of California Berkeley in 1966, and his PhD from the University of California San Diego in 1974. From 1974–77, he worked as a postdoctoral research associate in the laboratory of Gunther S. Stent* at the University of California Berkeley, where he identified a network of bilaterally paired, rhythmically active interneurons as components of the central pattern generator (CPG) that controls the swimming rhythm in the medicinal leech. He and Stent showed that recurrent cyclic inhibition plays a key role in the generation of the rhythmic neural burst activity of this CPG. In 1977, Friesen established his own laboratory at the University of Virginia, where he progressed through the faculty ranks to full professor. There, he continued to study neural mechanisms underlying rhythmic locomotion in the medicinal leech, including how swimming is modulated by sensory input. Friesen retired in 2013. Out of the 56 original papers that he published during his career, 22 appeared in JCPA.

**James Howard Fullard** (1952–2010) was born in British Columbia. He was an undergraduate the University of Toronto (1971–75) where he worked with Glenn K. Morris on the biology of insect auditory systems. He then moved to Carleton University where he received his master’s (1976) and his PhD (1979) degrees, on the topic of sound production in tiger moths, under the mentorship of M. Brock Fenton. He conducted postdoctoral research with Morris at the University of Toronto and with James A. Simmons at the University of Oregon. Fullard joined the faculty at the University of Toronto (1980), where he rose through the ranks to full professor (1993) and where he remained until his death. The James H. Fullard Nature Trial, near the Queen’s University Biological Station, was named in his memory. Over his career, Fullard travelled extensively throughout the tropics to conduct field work on insects and bat-insect interactions. Among his approximately 100 original research articles, he published 19 papers in JCPA. These papers describe in detail the auditory sensitivity of insects (tympanate moths, crickets) on the basis of acoustic, behavioral, and physiological measurements, and the behavioral strategies that have evolved in these species to avoid predation by echolocating bats.

**Howard Carl Gerhardt** (1945-) was born in Newport News, Virginia and grew up in Savannah, Georgia. He received his Bachelor of Science in zoology at the University of Georgia in 1966, and his PhD from the University of Texas at Austin in 1970. From 1970–71, he was a postdoctoral researcher with Robert R. Capranica* at Cornell University studying the neurobiology and behavior of amphibians. In 1971, he was appointed as an assistant professor at the University of Missouri, where he progressed through the faculty ranks to full professor. He is now a Curators’ Distinguished Professor Emeritus of Biological Sciences. Gerhardt received many honors and awards including the Senior Humboldt Award (1995), the Frank Beach Award (2001), the Distinguished Herpetologist Award from the Herpetologist League (2006), the Thomas Jefferson Award of the University of Missouri (2012), and the Quest Award from the Animal Behavior Society (2015). He is a Fellow of the Animal Behavior Society (elected 1991) and of the American Association for the Advancement of Science (1998). Gerhardt has contributed significantly to the understanding of the evolution and neurobiology of acoustic communication in amphibians. He identified the pertinent properties of male calls that are used by females for mate choice. By systematically varying the acoustic properties of synthetic sounds that mimic the stereotyped calls of males and by measuring the responses of females, he found that female grey treefrogs prefer the calls that are the most energetically expensive for the males to produce. This allows females to ensure that the male they select is not only in good physical condition, but also possibly genetically better than his neighbors. He co-authored with Franz Huber* the book *Acoustic Communication in Insects and Anurans* (Chicago University Press, 2002). Of the 207 publications (including books and book chapters) produced by Gerhardt, 20 highly cited papers appeared in JCPA.

**Martin Giurfa** (1962-, born in Lima) is a well-known neuroethologist and a pioneer in the field of insect cognition, with a special interest in the cognitive abilities of bees. Following an undergraduate degree in biology at the University of Buenos Aires, Giurfa obtained a PhD at the same university under the supervision of Josué Núñez. In 1990, he joined the laboratory of Randolf Menzel* at the Free University in Berlin where he built a reputation for his studies on color vision of bees. He obtained the *Habilitation* from the Free University of Berlin in 1997, and became assistant professor in the Institute of Neurobiology there. In 2001, Giurfa became professor of neuroscience at Paul Sabatier University in Toulouse where he continued his research on insect cognition. Since 2023, he has been Exceptional-Class Professor of Neurosciences at the Sorbonne University. He has been the recipient of numerous awards and fellowships, among them membership of the German National Academy of Science (Leopoldina; 2007), the Royal Academies for Science and Arts of Belgium (2018), the Silver Medal of the French National Center for Scientific Research (2007), and the International Prize of Science & Technology "Raices" of the Argentinean Government (2013). He is an editor of the volume *Honeybee Neurobiology and Behavior* (2012) with C. Giovanni Galizia and Dorothea Eisenhardt. Giurfa is particularly interested in understanding the principles underlying learning, memory and decision-making in bees, employing both behavioral and neurophysiological methods in his investigations. Of his eight papers published in JCPA, one in particular—on the rich behavioral repertoire of honeybees, and their remarkable capacity for complex learning and memory (Giurfa 2007)—has had a major influence on the field of insect cognition.

**Wulfila Gronenberg** (born 1954 in Berlin) is professor in the Department of Neuroscience, University of Arizona. Gronenberg studied biology at the Free University of Berlin and received his *Diplom* (master’s degree) in 1979. In his PhD thesis research, supervised by Joachim Erber* at the Free University of Berlin, he characterized neurons innervating the mushroom body of the honeybee brain. After receiving his PhD in 1984, he held various research positions, first with Friedrich G. Barth* at Goethe University Frankfurt working on mechanoreceptors in spiders, and then with Nicholas Strausfeld* at the University of Arizona, characterizing descending neurons in flies. From 1991–99, he was assistant and later associate professor with Bert Hölldobler at the University of Würzburg, focusing on the analysis of trap-yaw mechanisms in ants. In 1999, he became associate professor at the Arizona Research Laboratories, Division of Neurobiology. Since 2016, he is full professor at the Department of Neuroscience of the University of Arizona. Here, Gronenberg continued research on hymenopteran mushroom bodies by characterizing their sensory (especially visual) inputs and multimodal convergence. More recently, his interests have shifted to questions concerning brain development, evolution and plasticity in social insects. Of more than 80 articles published in journals and as book chapters, Gronenberg has contributed six highly cited publications to JCPA.

**Kurt Hamdorf** (1929–2009) was born in Hamburg. He studied biology at the University of Hamburg and joined the group of the marine biologist Adolf Bückmann for a *Diplom* (master’s) and PhD thesis. In 1958, he received his doctorate (*Dr. rer. nat.*) for studies on the role of light on the development of the rainbow trout. He then joined the group of Hansjochem Autrum* at LMU Munich and changed his focus of interest to study primary visual processes in insects. Following his *Habilitation* at LMU Munich, Hamdorf was appointed in 1969 as professor of visual physiology at the Ruhr-University of Bochum where he stayed until his retirement in 1994. Hamdorf contributed significantly to furthering our understanding of primary visual processes and the mechanisms of phototransduction in insects. In addition, he also contributed to research on visually triggered landing responses in flies. Hamdorf’s 31 publications in JCPA focus on absorption properties of visual pigments, mechanisms of phototransduction, visual adaptation, spectral sensitivity, pupil mechanisms, and photoregeneration in flies and moths.

**Roger Clayton Hardie** (1953-) studied at the Australian National University with George Adrian Horridge*, and received his PhD in 1979 for studies on peripheral visual function in flies. After completing his PhD, he moved to the Max Planck Institute for Biological Cybernetics in Tübingen as a postdoctoral fellow. In 1986, he moved to the University of Cambridge, where he remains a professor today. He was elected a Fellow of the Royal Society (2010) and was awarded the Rank Prize in Optoelectronics for his outstanding contributions to research on the physiology of insect vision. One of Hardie’s first papers, revealing common strategies for light adaptation in the peripheral visual systems of flies and dragonflies, was published in JCPA in 1978 and cited 329 times. In addition, a series of four highly cited papers, dating back to his dissertation, in which he characterized the fly retina electrophysiologically, were published in JCPA (1979). Hardie characterized the sex-specific photoreceptors in the anterior dorsal eye of male houseflies by microspectrophotometry and intracellular recordings, and he studied the pigment system of photoreceptor cell R7 in flies. Furthermore, he showed that histamine is the neurotransmitter released by photoreceptors and that it directly activates ion channels on adjacent cells in the visual pathway. Again, these articles were published in JCPA, and the histamine article alone is cited 344 times. He identified genes in fruit flies that encode transient receptor potential ion channels through which calcium flows in response to light, and he characterized the visual phototransduction cascade in flies, including the role of arrestin. In total, Hardie published 17 original research articles in JCPA (out of his approximately 200 articles).

**Nathan Hart** (1973-) is professor and head of the School of Natural Sciences at Macquarie University. Hart was educated during the 1990s at the University of Bristol (BSc and PhD), and moved to Australia in 1999, first as a postdoctoral researcher at the University of Queensland and then as an associate professor at the University of Western Australia. He is particularly interested in the evolution of vertebrate color vision, and addresses this question in ‘early’ vertebrates such as lampreys and sharks. He also studies the color vision of birds to understand how this visual modality is used in their sexual and natural selection. His highly cited papers in JCPA all deal with the color vision of birds, and in particular how the absorption spectra of the visual pigments and the overlying oil droplets affect color perception and are related to the ecologies of different species. In more recent times, he has devoted his research efforts to developing methods that deter sharks and protect humans from shark bites.

**Walter Heiligenberg** (1938–1994; for obituaries see Zupanc and Lamprecht 1994; Zupanc and Bullock 2006) was born in Berlin. After two years of undergraduate study in zoology and botany at the University of Münster, he conducted his PhD thesis research under the guidance of Konrad Lorenz and Hansjochem Autrum* at the Max Planck Institute for Behavioral Physiology in Seewiesen from 1960–63. In this study, he analyzed the influence of motivational factors on the occurrence of behavioral patterns in cichlid fish. During the following nine years, he continued his behavioral studies at the same institute. A widely recognized achievement during this time was his quantitative demonstration of the phenomenon of heterogeneous summation. In 1972, he joined the Neurobiology Unit at the Scripps Institution of Oceanography of the University of California San Diego, headed by Theodore H. Bullock*. One year later, he established his own laboratory there. His trainees include Gary Rose* and Günther Zupanc*. During the following two decades, Heiligenberg succeeded in what Rüdiger Wehner* once characterized as “one of the best, if not *the* best and most complete case study available in neuroethology” (Wehner 1994), namely his work on the jamming avoidance response in the weakly electric fish *Eigenmannia*. Together with his associates, he deciphered some of the key computational rules and many of the details of the neural network underlying this behavior. Heiligenberg had a very close relationship with JCPA—not only as an associate editor but also as one of its most prolific authors. Of the 170 publications (including books and book chapters) authored by himself and/or members of his lab, 59 appeared in JCPA. On 40 of them, Heiligenberg is listed as (co-)author. His book *Neural Nets in Electric Fish* (MIT Press, 1991) provides a comprehensive summary of his research on the jamming avoidance response and the underlying neural network. He died at the zenith of his scientific career when he was aboard an aircraft that crashed near Pittsburgh, Pennsylvania.

**Martin Heisenberg** (1940-) was born in Göttingen and studied chemistry and molecular biology at the University of Tübingen. In 1966, at the age of 26, he completed his doctoral thesis on a topic concerning the genetics of bacteriophages. He then went to the California Institute of Technology (1966–68) as a postdoctoral researcher in the laboratory of Max Delbrück, where he learned molecular biology methodologies. He returned to Germany in 1968 to assume an assistant professorship with Karl-Georg Götz at the Max Planck Institute for Biological Cybernetics in Tübingen, where he began his research on the fruit fly. In 1975, Heisenberg took over the Chair of Genetics and Neurobiology (at that time still Chair of Genetics and Microbiology) at the University of Würzburg. He held this chair for almost 35 years until his retirement in 2009, when he accepted a senior professorship at the Rudolf Virchow Centre in Würzburg. Heisenberg was elected as a member of the German National Academy of Sciences (Leopoldina) in 1989, and of the Academia Europaea in 1988. Included among his many awards are the Karl Ritter von Frisch Medal (2006) and the Röntgen-Medal (2015). He was president of the Society for Neuroethology from 2007–10. During his many years at the University of Würzburg, Heisenberg, as one of the world’s leading neurobiologists and geneticists, made outstanding contributions to the field of visual behavior, and learning and memory. He was one of the first to use brain development mutants in fruit flies to study the relationship between brain structures and behavior, and is considered the founder of neurogenetics in Germany. Out of his approximately 182 research articles, 12 highly cited papers on visual behavior appeared in JCPA.

**Roland Hengstenberg** (1940–2004) was born in Esslingen, Germany and studied biology, chemistry, geography, and philosophy at the Christian-Albrechts-University Kiel, the Albert-Ludwigs-University Freiburg, the University of Stuttgart, and the University of Tübingen. In 1963, he passed the first state examination for teaching at high schools but was clearly more inclined to research than to teaching. In 1971, he earned his PhD in zoology, botany, and chemistry at the University of Tübingen and then pursued postdoctoral training at the Cold Spring Harbor Laboratories. Consecutively, he worked as a scientist at the Max Planck Institute for Biological Cybernetics, Tübingen. From 1976–79 he was member of the Scientific Council of the Max Planck Society and from 1981–82 he visited the Australian National University. Hengstenberg devoted his research to gaze stabilization systems that have evolved during evolution in all animal groups with high mobility and good vision. He investigated the eye movements in flies that are necessary to reduce the image speed on the retina enabling acceptable vision even during faster body movements and identified the neurons that are responsible for extracting specific rotational movements from the optic flow field during flight as well as the mechanisms that keep flight stable. Furthermore, he worked with Gerbera Nalbach* on the halteres of the blowfly. These results were published in six highly cited articles in JCPA that appeared from 1982–94.

**Horst Hertel** (1945-) was born in Groß-Gerau, Germany. From 1966–71, he studied biology at the Technical University of Darmstadt. For his diploma thesis and dissertation supervised by Randolf Menzel*, he studied the spectral sensitivity of a rotifer and characterized the compound eye of brine shrimps for which he received his *Dr. rer. nat.* in 1976. In 1976, together with Menzel, he moved to the Free University of Berlin and as research associate, and later assistant professor, focused on the mechanisms of color vision in honeybees using intracellular recordings. He received his *Habilitation* in 1988. In 1987, Hertel took a position at the Federal Institute for Materials Research and Testing in Berlin, while still remaining adjunct professor at the Free University. At the Federal Institute he became director of the Expert Group in Biology and was involved in developing tests for and protection measures against wood-destroying organisms such as termites, beetles and fungi. These activities included basic research, including behavioral studies on relevant species such as termites and the European house borer. Hertel retired in 2010. He has authored six highly cited articles in JCPA on peripheral and central mechanisms of color vision in honeybees.

**John G. Hildebrand** (1942-), born in Boston, is the international secretary of the National Academy of Sciences (US) and Regents Professor Emeritus of Neuroscience and Honors Professor at the University of Arizona. He earned his BA in biology (1964) at Harvard University, mentored by John Law and Konrad Bloch, and his PhD in biochemistry (1969) at Rockefeller University, mentored by Leonard Spector and Fritz Lipmann. Hildebrand pursued postdoctoral work in neurobiology (1969–71) with Edward A. Kravitz at Harvard Medical School. There he studied the operation and distribution of neurotransmitters in the lobster nervous system. Hildebrand held faculty positions at Harvard Medical School (1976–80) and at Columbia University (1980–85) before joining the University of Arizona in 1985 as the founding Director of the Arizona Research Laboratories, Division of Neurobiology. Here, he created a unique and highly acclaimed center for neuroscience focusing on insect nervous systems. Two of his trainees (Thomas Christensen* and Uwe Homberg*) are included here among the Top 100 Authors. He remained at the University of Arizona as professor of neuroscience, chemistry & biochemistry, ecology & evolutionary biology, entomology, and molecular & cellular biology until his retirement in 2022. Hildebrand is an elected Fellow of the American Academy of Arts and Sciences (2001), American Association for the Advancement of Science (1986), American Philosophical Society (2014), Entomological Society of America (2008), International Society for Neuroethology (2012), and the Royal Entomological Society of London (2012). He is a member of the US National Academy of Sciences (elected 2007) and the German National Academy of Sciences ‘Leopoldina’ (1998), and a foreign member of both the Norwegian Academy of Science and Letters (1999) and the Royal Norwegian Society of Sciences and Letters (2011). He served as president of the International Society for Neuroethology (1995–98) and as an associate editor of JCPA (1990–2021). He is also known as an accomplished trombonist, and once considered a professional career in music. Hildebrand’s research program focuses on chemosensation in insects, particularly the giant sphinx moth. His work exploits and integrates anatomical, behavioral, chemical, molecular, and neurophysiological methodologies to elucidate the molecular biology, physiology, and function of olfactory behaviors. His 22 papers in JCPA present new findings on sex pheromones, chemical preferences, odorant receptors, interneuron diversity, and antennal lobe morphology in beetles and moths. A tribute to Hildebrand and his work, published in JCPA in form of a Special Issue entitled *Insect Chemoreception* (Volume 199, Issue 11, 2013), was edited by Wolfgang Rössler and Monika Stengl.

**Uwe Homberg** (1953-) was born in Wuppertal, Germany. From 1972–78, he studied biology, first at the Technical University of Hannover, then at the Free University of Berlin where he completed his *Diplom* (master’s) thesis in the lab of Randolf Menzel*, supervised by Joachim Erber*. He then stayed on at the Free University and performed his PhD thesis research under the mentorship of Erber. After the award of a PhD in 1982, Homberg assumed various academic positions at German and U.S.-American institutions, including the Technical University Berlin, Columbia University, the University of Arizona, the University of Konstanz (where he received his *Habilitation* in zoology in 1992), and the University of Regensburg. During these years, his work in the laboratory of John G. Hildebrand* was particularly formative. In 1997, Homberg was appointed to a professorship at the Philipps University of Marburg, from which he retired in 2021. Throughout his scientific career, his research was guided by the goal to decipher the general principles of the structure, function, and development of the insect brain. Major topics have included elucidation of sensory control of spatial orientation and navigation; neural analysis of polarization vision; exploration of mechanisms of circadian rhythms; investigations of structure and function in olfactory systems; and brain mapping of neurotransmitters and neuropeptides. He has frequently addressed these aspects within a comparative framework. The success of his research program is reflected by more than 170 original papers, review articles, book chapters, and books, and by the over 12,000 citations that these publications have attracted. Homberg has been a member of the Editorial Board of JCPA since 2012. In addition to 19 journal articles, his contributions to JCPA include the editing of two Special Issues, one entitled *Visual Circuits in Arthropod Brains* (Volume 206, Issue 2, 2020) and a second, jointly edited with Keram Pfeiffer, titled *Unravelling the Neural Basis of Spatial Orientation in Arthropods* (Volume 209, Issue 4, 2023).

**George Adrian Horridge** (1927-, born in Sheffield, UK) is emeritus professor of neurobiology (Research School of Biological Sciences) at the Australian National University. Horridge is a foundational figure in the field of insect vision, and the research environment he created in Australia was the internationally leading center in this field for over three decades. Horridge was educated at the University of Cambridge, first as an undergraduate and then as a postgraduate (earning a PhD there in 1953). He was appointed a Fellow of St. John’s College Cambridge in the same year. From 1960–69, he was the director of the Gatty Marine Laboratory at the University of St. Andrews, before becoming a founding professor at the newly established Research School of Biological Sciences at the Australian National University in 1969. During his early years at the Gatty Marine Laboratory, Horridge teamed up with Theodore H. Bullock* to write the monumental two-volume “bible of invertebrate neurobiology”, *Structure and Function in the Nervous System of Invertebrates* (Freeman, 1965), which remains a classic today. Horridge has been the recipient of numerous honors, among these Fellowships of the Royal Society of London, the Australian Academy of Science (1971), and the Centenary Medal from the government of Australia (2001). He has also been awarded a Centenary Medal “for service to Australian society in the biological sciences”. The list of his trainees and collaborators comprises many distinguished scientists, including 10 of the Top 100 Authors—Roger Clayton Hardie*, Almut Kelber*, Michael Land*, Simon Laughlin*, Randolf Menzel*, Daniel Osorio*, David C. Sandeman*, Mandyam V. Srinivasan*, Doekele Stavenga*, and Eric J. Warrant*. After retirement, Horridge became interested in the visual system of the honeybee, and performed a large number of behavioral experiments on bees in his garden. This work has resulted in several papers in JCPA and three books: *The Discovery of a Visual System: The Honeybee* (CABI, 2019), *Honeybees Vision: Recent Discoveries* (Northern Bee Books, 2021) and *How do Bees (and Humans) see Grey Levels?* (Northern Bee Books, 2023).

**Ronald R. Hoy** (born 1939, Walla Walla, Washington) is Dr. David and Dorothy Joslovitz Merksamer Professor of Biological Science Emeritus at Cornell University. He received his undergraduate degree in zoology and psychology (1962) at Washington State University where he worked with Leonard Kirschner. His graduate training took place at Stanford University, where he received his PhD in biology (1968) under the direction of Donald Kennedy. Hoy’s dissertation research used neurophysiological techniques to examine degeneration and regeneration in crayfish motor neurons. He pursued postdoctoral studies with David Bentley at the University of California Berkeley (1969–71), studying the development of neural circuits in crickets and the neurobiology of cricket song. In 1973, Hoy joined the faculty at Cornell University (Department of Neurobiology and Behavior), where he spent the rest of his academic career. In 2002, he was named Howard Hughes Medical Institute Professor, a position he held until 2006. Hoy’s laboratory exploited behavioral and electrophysiological techniques to analyze sensory processing and communication in insects, including several species of crickets, spiders, treehoppers, flies, and mosquitos. Based on this work, he designed and patented a directionally-sensitive miniature microphone with the potential to improve hearing aids for humans. Hoy is committed to teaching. Even after retiring from Cornell in 2021, he continues to offer courses such as ‘Music and the Brain’, drawing on his scientific expertise and his long-standing interest in music. He and his collaborators developed CRAWDAD, a series of laboratory exercises for undergraduate students using crayfish as a model system for teaching neurophysiology. For several years (1979–84), Hoy directed the Neural Systems and Behavior course at the Marine Biological Laboratory. He was honored with the Neuroscience Educator of the Year award (2004) from the Association of Neuroscience Departments and Programs, and a Lifetime Award for Contributions in Neuroscience Teaching (2006) from the Society for Neuroscience. Hoy is a Fellow of the American Association for the Advancement of Science (1986) and of the American Academy of Arts and Sciences (2010). He is a lifetime trustee of the Grass Foundation for Neurobiology and of the Cornell Laboratory of Ornithology. His 20 publications in JCPA include behavioral and neurophysiological studies of ultrasound-induced acoustic startle in crickets as a countermeasure to predation by echolocating bats, the use of phonotaxis to examine acoustic cues necessary for species recognition, directional sensitivity of insect hearing and vibratory organs, temperature coupling in cricket communication, and the use and processing of visual signals by jumping spiders.

**Franz Huber** (1925–2017; for an obituary see Barth 2017) was born in Nussdorf near Traunstein, Germany. He grew up on a farm, where animals were an integral part of his life. In 1947, he enrolled at LMU Munich to study biology, chemistry, and physics. Five years later, he completed his doctoral thesis under the mentorships of Werner Jacobs and Karl von Frisch*. In his thesis research, he succeeded in eliciting complex song patterns by applying lesions to specific, tiny areas of the brain in the field cricket— then a groundbreaking achievement. Subsequently, as a scientific assistant of Franz Peter Möhres at the University of Tübingen, he was the first to conduct focal brain stimulation experiments in insects. He had learned this rather novel technique during a research visit to the University of Zurich in 1956, where Walter Rudolf Hess had developed it for his famous studies of the functional organization of the feline diencephalon. Brain-stimulation and brain-lesioning experiments also formed the basis for Huber’s *Habilitation* at the University of Tübingen in 1960. Other scholars who had a lasting influence on Huber were Kenneth Roeder of Tufts University, who had paved the path to relating nervous system function to behavior in insects, and Theodore H. Bullock*, from whom he learned intracellular recording techniques during a visit to the University of California Los Angeles. In 1963, Huber was appointed to a full professorship at the University of Cologne, and in 1973 he became director at the Max Planck Institute for Behavioral Physiology in Seewiesen, where he remained until his retirement in 1993. His accomplishments have been recognized by numerous awards, including the Karl Ritter von Frisch Medal of the German Zoological Society. He was a member of the German National Academy-Leopoldina, the Academia Europaea, the Bavarian Academy of Sciences, the American Academy of Arts and Sciences, and the German Zoological Society. In 2016, he was elected a Fellow of the International Society for Neuroethology. The integrative approach employed by Huber and his students pioneered the study of neural control of behavior at the level of single nerve cells and neural networks in insects. Several of his books have become must-reads for neuroethologists, including *Cricket Behavior and Neurobiology* (together with Thomas Edwin Moore and Werner Loher, Cornell University Press, 1989) and *Acoustic Communication in Insects and Anurans* (together with H. Carl Gerhardt*, Chicago University Press, 2002). Huber published 19 papers in JCPA; their impact is reflected by the well over 1600 citations they have attracted.

**Philip H.-S. Jen** is professor emeritus of biological sciences at the University of Missouri. Born in Taiwan, he moved to the US to study auditory neuroscience with Nobuo Suga at Washington University. Jen received his PhD in 1974. His graduate research examined the tonotopic organization of the auditory cortex in constant-frequency echolocating bats, and described how Doppler shifts are encoded in the bat’s peripheral and central auditory systems. From 1982 until his retirement in 2012, Jen was a faculty member in the Division of Biological Sciences at University of Missouri. He was also affiliated with Central China Normal University (2001–12) and National Taiwan Normal University (2008–09), and is currently conducting studies at Binzhou Medical University on hearing sensitivity in humans. Jen is an elected Fellow of the American Association for the Advancement of Science (2001). He was an early investigator of descending input to the inferior colliculus from the cortex, which affected subsequent ascending signals. His 24 papers in JCPA describe the neurophysiology of hearing and echolocation in frequency-modulated echolocating bats. This work uncovered how the auditory midbrain and cortex code the direction, spatial position, frequency structure, and duration of complex echolocation signals, how these processes are affected by inhibition, and how the cortex modulates activity in the midbrain.

**William T. Keeton** (1933–1980) was born in Roanoke, Virginia (for an obituary see Emlen 1981). He attended the University of Chicago from which he received both bachelor of arts and bachelor of science degrees. He then pursued a master’s degree in entomology at Virginia Polytech Institute. In 1956, Keeton arrived at Cornell University, where he worked under Howard E. Evans on millipede systematics. Upon earning his PhD in 1958, he joined the faculty of the Department of Entomology at Cornell. During the following years, he established his reputation as a leading expert in milliped research. In 1965, he moved from the Department of Entomology into the Section of Neurobiology and Behavior, which he had helped create, and where he trained Melvin Kreithen*. Building upon his childhood hobby—keeping and racing homing pigeons—Keeton developed a vigorous research program for exploring the behavioral processes that enable pigeons to find their way home. As a milestone achievement, he discovered that under overcast skies, when celestial cues are no longer available for orientation, pigeons can still home. Keeton obtained experimental evidence that geomagnetism was involved in this ability. He demonstrated that test pigeons perform much more poorly than control pigeons in selecting the homeward direction when small bar magnets were attached to their bodies before they were released. This discovery not only opened new avenues for research into magnetoreception and magnetic orientation but also indicated the inherent difficulty of revealing orientation mechanisms in migrating and homing animals by the presence of multiple orientation systems with substantial redundancies. Besides his pioneering work on animal navigation, Keeton was also a popular teacher at Cornell. He spent several years of his career translating his profound expertise as a lecturer into a textbook, *Biological Science* (W.W. Norton). Between its first publication in 1967 and his death in 1980, this textbook appeared in three highly successful editions. The 10 papers that Keeton published in JCPA have attracted on average over 70 citations per article, underscoring the impact of his work on the research of his peers.

**Almut Kelber** (1962-) is currently director of Research Grants at the Human Frontiers Science Program in Strasbourg, and an adjunct professor of sensory biology at the Lund Vision Group at the University of Lund. After her education in Germany (at the University of Tübingen for both her undergraduate degree in 1989 and her PhD in 1993), Kelber pursued postdoctoral training with George Adrian Horridge* at the Australian National University (where she studied butterfly color vision). She then moved to the Lund Vision Group in Sweden to work on nocturnal vision in moths and bees with Eric Warrant* (in 1999). In Lund, Kelber soon established an independent group, eventually becoming professor and finally research dean at the Faculty of Science. She joined the Human Frontiers Science Program in 2020. She is very well known for her influential work in comparative color vision, particularly in butterflies, moths, amphibians and birds, in which she has combined quantitative behavioral experiments to elucidate the color vision abilities of the animals she studies, with rigorous modelling of the underlying visual signals generated in the different spectral classes of photoreceptors. Kelber is particularly well known for her work on color vision in dim light, and for discovering the first nocturnal animal known to have color vision (a nocturnal hawk moth). Her many well-cited papers in JCPA reveal the breadth of her research interests, with papers on color vision, spatial vision and temporal vision in both birds and insects, as well as papers on how visual and olfactory cues are used by insects during foraging.

**Manfred Kössl** (1958-), born in Munich, is Head of Neurobiology and Biological Sensors at Goethe University Frankfurt. He studied biology and physics at LMU Munich and the University of Tübingen from 1977–87. His doctoral thesis examined frequency processing in the peripheral auditory system of bats. With support from the German Research Foundation, he undertook postdoctoral training (1988–90) with Ian R. Russell at the University of Sussex, studying hair cell physiology. From 1990–97, Kössl worked as a research assistant at the Zoological Institute of LMU Munich. He completed his *Habilitation* in neurobiology in 1994. In 1997, he became Heisenberg Fellow of the German Research Foundation at the Zoological Institute of LMU Munich. From 1991–2006, he took yearly trips to Cuba and to Jamaica to study bats. Kössl became professor of neurobiology at the Zoological Institute of Goethe University Frankfurt in 2001. There he established a diverse and productive auditory research group that undertakes broad, comparative behavioral and neurobiological analyses of bat echolocation and of insect hearing. He served as director of the Centre for Interdisciplinary Neuroscience at Goethe University from 2012–18. Kössl’s 28 papers in JCPA describe cochlear organization and the acoustic fovea in constant-frequency bats, cochlear sensitivity and active hearing in insects (moths, crickets, locusts), the development of objective methods for quantification of auditory brainstem responses, evolutionary adaptations for echolocation and for insect countermeasures, and the functional organization of the central auditory system in several species of bats for processing spectral and temporal properties of echolocation sounds.

**Melvin L. Kreithen** (1941–1997) was born in Philadelphia. He received a bachelor’s degree from the University of Maryland (1968) and, under the mentorship of William T. Keeton*, a PhD from Cornell University (1974). He remained at Cornell as a research associate (1974–80), then accepted a faculty position in biology at the University of Pittsburgh (1980), where he remained until his death. He was a member of the American Racing Pigeon Union (1992–97) and was on the advisory panel of United States Windpower (1992–97). Kreithen’s research focused on the identification and quantification of sensory cues used by pigeons and by monarch butterflies to control navigation, homing, orientation, and migration. He was involved in efforts to limit hazards to flying birds due to aircraft and wind turbines, and held a patent for a novel deterrent device. Kreithen published six papers in JCPA. Some of these used operant conditioning methods to demonstrate that homing pigeons detect polarized light, atmospheric infrasound, and changes in magnetic cues. He also examined the role of olfactory cues in pigeon homing behavior.

**Michael Land** (1942–2020; for an obituary see Marshall and Cronin 2021) was a foundational figure in comparative physiological optics and the study of eye movements in animals. He was educated at the University of Cambridge (with a degree in zoology in 1963) and he completed a PhD at University College London in 1968. Following an assistant professorship at the University of California Berkeley, Land returned to the UK in 1971, helping John Maynard Smith establish a new Department of Neurobiology at the University of Sussex. Land eventually became a professor there in 1984, and trained, among others, Jochen Zeil*. He has received numerous honors, among these fellowships of the Royal Society of London (at the age of 39, one of the youngest elected in the Society’s history), and the Acadaemia Europaea (1998). He was awarded the Rank Prize ( 1998), and elected as Fellow of the International Society for Neuroethology (2012). Land’s work revealed the remarkable optical mechanisms that have evolved in animal eyes, and was responsible for describing several previously unknown eye types in nature. He worked mostly on invertebrates, discovering the concave mirror eyes of bivalve mollusks, the reflecting superposition eyes of decapod crustaceans, the unusual moveable retinas of jumping spider principal eyes, and the afocal optics of butterfly apposition eyes (with Dan-Eric Nilsson and Joe Howard). His work also revealed how the basic optical designs of eyes can be molded by evolution to match animals to their lifestyles and habitats, a field known as ‘visual ecology.’ Land’s work on the optical mechanisms of eyes—much of it published in a large number of highly cited papers in JCPA—was paradigm-shifting and revealed the stunning variety of eyes that have evolved on our planet. His work culminated in two classic books on the eyes of animals: *Animal Eyes* (with Dan-Eric Nilsson; Oxford University Press, 2012) and *Eyes to See* (Oxford University Press, 2018). His later work shifted to the mechanisms and functions of eye movements in animals (including humans), with this work also resulting in an acclaimed book on the subject: *Looking and Acting: Vision and Eye Movements in Natural Behaviour* (together with Benjamin Tatler; Oxford University Press, 2009).

**Gerald D. Langner** (1943–2016) graduated with a *Diplom* (master’s) degree in physics from the Technical University of Munich (1971). He received his PhD at the Max Planck Institute for Biophysical Chemistry in Göttingen, under the direction of Otto Creutzfeldt. In 1975, he joined the laboratory of Henning Scheich* at the Technical University of Darmstadt. During a research visit to the Australian National University (1985), Langner and Scheich discovered that the bill of the platypus is an electroreceptive organ, and that these animals can locate objects by sensing electric fields. Langner was appointed to a professorship in neurobiology at the Technical University of Darmstadt in 1988, a position he held until his retirement in 2008. One year before his death, he published *The Neural Code of Pitch and Harmony* (Cambridge University Press, 2015), in which he proposed a temporal-based neural mechanism of music perception. Langner’s eight papers in JCPA quantify responses of central auditory neurons in chicks, guinea fowl, and gerbils to complex sounds including species-specific vocalizations. He also contributed a study of sound sensitivity in the human auditory cortex based on magnetoencephalography.

**Simon Laughlin** (1947-) is emeritus professor of neurobiology, and professorial Fellow of Churchill College, University of Cambridge. He was educated at the University of Cambridge (undergraduate degree in zoology in 1967) and the Australian National University (PhD 1974), where he studied with George Adrian Horridge*. Laughlin stayed at the Australian National University until 1984, when he returned to the University of Cambridge. He became professor of neurobiology there in 2004. Laughlin was awarded a Rank Prize Research Professorship in Optoelectronics (1999–2004) for work on the biological processing of images, and a Distinguished Fellowship at the Sage Center for the Study of the Mind at the University of California Santa Barbara (2019). He is a Fellow of the Royal Society of London (2000). He is renowned for his work to uncovering the principles of early visual processing, particularly in insects, and how these principles are matched to the ecologies of insects. Throughout his career, he has been fascinated by how the acquisition of visual information is shaped by the statistical properties of natural scenes and constrained by the optical and neural designs of the eye and its visual cells (with several well-cited papers in JCPA on these topics). He was the first to ask how much visual information actually costs in terms of the number of ATP molecules expended to acquire it, and showed that this expenditure ultimately limits the size of the eye and thus plays a major role during the evolution of vision (and indeed of other senses). Many of these ideas were summarized in his acclaimed book, *Principles of Neural Design* (together with Peter Stirling; MIT Press, 2015).

**Martin Lindauer** (1918–2008; see obituary by Wehner 2009 and a laudation on his 80th birthday by Barth 1999) was a leading light in the discovery of how honeybees communicate and learn, sense the world, find their way, and live in societies. He was born in a small village in the foothills of the Bavarian Alps as the 11th of 15 siblings. Shortly after his successful *Matura* (high school diploma) degree in 1939, the Second World War broke out and he had to join the army. In 1942, he was released from the army after suffering a severe injury and then started to study biology at LMU Munich. He became a PhD student of Karl von Frisch* from 1945–47 and then his scientific assistant, first in Graz and from 1950 onward in Munich until von Frisch retired in 1958. In 1963, Lindauer accepted an offer from Goethe University Frankfurt and worked there as a professor at the Zoological Institute until 1973, when he joined the University of Würzburg. He remained in Würzburg until his retirement in 1987. Lindauer has been awarded many honors, including honorary doctorates from the Universities of Zürich, Umeå, and Saarbrücken, the Order of the Federal Republic of Germany first class, and the Bavarian Maximilian Medal. He was a member of the American Academy of Arts and Sciences (1962), the American Philosophical Society (1976), and the US National Academy of Sciences (1976). For many years, Lindauer had a close relationship to JCPA, not only as author but also as co-editor. He published 20 papers in JCPA. The topics of sensory and behavioral biology were always in the forefront of the journal and of his research interest, especially the topics of communication among bees and their orientation. Research on temperature regulation, water balance and division of labor in the bee colony, orientation with the help of the solar compass, visual perception of form, perception of the Earth's magnetic field, sense of smell, gravity perception, and the ability of the honeybee to learn and remember would be unthinkable without Lindauer's work.

**Klaus Lunau** (1953-) is professor emeritus at the University of Düsseldorf. He studied biology at the University of Freiburg. Following projects in nature conservation, he joined the group of Günther Osche in Freiburg and received his doctorate (*Dr. rer. nat*.) in 1988 studying innate preferences of bumblebees for optical flower signals. He then took a position as research and teaching associate with Dietrich Burkhardt at the University of Regensburg, focusing on insect flower interactions and eye specializations in flies. He received his *Habilitation* from the University of Regensburg in 1995. In 1997, Lunau was appointed full professor of zoology at the University of Düsseldorf. His interest continued to be the sensory ecology of insects, insect-plant interactions, and the evolution of floral traits in animal-pollinated plants, now with a focus on camouflage, mimicry, and odor collection by orchid bees. Lunau published several popular books on these topics as well as 13 highly cited articles on insect eye adaptations in JCPA.

**N. Justin Marshall** (1962-) was educated at the University of St. Andrews (BSc 1985) and the University of Sussex (PhD 1991), where he studied with Michael Land*. He has been at the University of Queensland since 1996. He is a distinguished neuroethologist and Laureate Fellow of the Australian Research Council at the University of Queensland. Marshall has received a number of prizes and fellowships, including election as Fellow of the Australian Academy of Science (2020), the L’Oreal Art and Science Award (2001), the ARC Discovery Outstanding Researcher Award (2012), an ARC Laureate Fellowship (2014), the IEEE Donald Fink award for bioinspired engineering (2016) and the Rank Prize for Optoelectronics (2020). He has made a reputation for his work on vision and visual ecology in the marine environment, and is particularly interested in how vision has been matched to different marine habitats (e.g., shallow and brightly lit colorful reefs or the dim blue featureless world of the deep sea), and to the different visual needs of animals (e.g., in the context of mating, defense and predation). He is best known for his discovery of the world’s most complicated color vision system—the 12-channel color vision of the mantis shrimp, an animal he has continued to study for his entire career. Marshall has also made major contributions to our understanding of color vision in fishes, cephalopods and birds and has made seminal discoveries in the use of polarized light in the marine environment, including the discovery of circularly polarized light sensitivity in the eyes of mantis shrimps. Much of this work is published in well-cited papers in JCPA. Apart from his research, Marshall has been a major advocate for the protection of coral reefs around the world, initiating a highly successful citizen science program (Coral Watch) devoted to monitoring reef health internationally.

**Michael Menaker** (1934–2021) was born in Vienna and grew up in New York City. After graduating from Swarthmore College in 1955, he joined the laboratory of Colin Stephenson Pittendrigh* at Princeton University. There, he did his PhD on endogenous circadian rhythms of bats and subsequently studied hibernation patterns in bats as a postdoctoral fellow in the lab of Donald R. Griffin (from 1959–62). In 1962, Menaker joined the University of Texas Austin and transitioned to studying circadian rhythms in the house sparrow and the golden hamster. In 1979, he moved to the University of Oregon, where he served as director of the Institute of Neuroscience. In 1987, he was recruited as chairman of the Department of Biology at the University of Virginia, where he remained as Commonwealth Professor of Biology until his retirement in 2020. Among his many awards were a Guggenheim Fellowship (1971–72), Fellow of the Japan Society for the Promotion of Science (1992), Fellow of the American Academy of Arts & Sciences (1999), Lifetime Achievement Award from the American Society of Photobiology (2002), and the Peter C. Farrell Prize in Sleep Medicine (2007). Menaker was one of the pioneers in the physiological analysis and identification circadian pacemakers in the vertebrate nervous and endocrine systems. His laboratory discovered nonretinal photoreceptors for entrainment in birds and reptiles; the first circadian structure controlling rhythmic behavior in vertebrates (the pineal gland of birds); the first single‐gene circadian mutation in mammals that affected the circadian clock (hamster *tau* mutation); and the existence of widespread circadian oscillators in peripheral tissues in mammals. Of the approximately 200 publications produced by himself and/or members of his lab, 22 appeared in JCPA.

**Randolf Menzel** (1940-) was born in Marienbad, Czech Republic. Menzel studied biology, chemistry and physics at the University of Tübingen and Goethe University Frankfurt. In 1967 he received his doctorate (*Dr. rer. nat.*) supervised by Martin Lindauer* at Goethe University Frankfurt for his research on color vision and learning in honeybees. Fascinated by the social organization and communication in a bee colony, he devoted his further scientific career to the study of honeybees, their visual and olfactory systems and, in particular, neural mechanisms of learning and memory. Following short postdoctoral positions with Lindauer at Goethe University Frankfurt and with Hubert Markl at the Technical University of Darmstadt, he received his *Habilitation* in 1971 and was appointed full professor at the Technical University of Darmstadt in 1972. In 1976, he accepted a position at the Free University of Berlin where he has remained, interrupted by research visits in Brazil, Australia, Israel, and the United States. Currently, he is emeritus professor there. Menzel has received numerous awards, including several honorary doctorates, the Leibniz Prize of the German Research Foundation (1991), Karl Ritter von Frisch Award (2004), and the International Prize of the Fyssen Foundation (2007). He is a member of the Academia Europaea and the German National Academy of Sciences (Leopoldina). Menzel has contributed groundbreaking work to the understanding of memory formation in the honeybee brain, their navigational skills and sensory processing. Hallmarks of his research are creative and innovative approaches to study brain function, navigational mechanisms, and cognition, that were highly influential throughout and beyond his field of research. Of his more than 250 articles and several books, he published 41 papers on navigation, learning and memory of the honeybee in JCPA, which together received the highest absolute number of citations of all authors.

**Jürgen J. Milde** (1950–2013) was an insect neuroscientist working on the visual system of flies, bees, and moths. Milde studied biology at the Free University of Berlin. He joined the group of Randolf Menzel* and received his doctorate (*Dr. rer. nat.*) in 1982 for work on intracellular recordings from ocellar interneurons in honeybees. For postdoctoral training, he joined the group of Nicholas Strausfeld* at the European Molecular Biology Laboratory in Heidelberg. There, he characterized visual interneurons and descending neurons in the brain of blowflies involved in optomotor responses. In 1988, he accepted a position as research associate with Gernot Wendler at the University of Cologne, where he characterized visual interneurons and the flight motor system of moths, and where he also received his *Habilitation*. In addition to his focus on visual processing, he contributed to research on leg reflexes in a wandering spider and on the role of adipokinetic hormone in a sphinx moth. In 1996, he left science for a position in the German Social Accident Insurance Institution. Milde published seven highly cited articles in JCPA.

**Nicolas Mrosovsky** (1934–2015) was born in Romania and educated at Winchester College. He received his MA from Magdalene College, University of Cambridge, and his PhD from University College London. In 1967, he joined the Department of Zoology at the University of Toronto, where he spent his entire career. He was awarded a Guggenheim Fellowship (1973), Canada Council Killiam Research Fellowship (1994), and the Lifetime Achievement Award from the Sea Turtle Society (2008). He was a Fellow of the Royal Society of Canada (1993). Mrosovsky had two main research interests that he pursued throughout his entire life: sea turtle biology and chronobiology. He came to his work on sea turtles through his initial interest in the phototaxis of frogs and turtles, and this work eventually led him to conservation biology. His interest in chronobiology stemmed from his early work on homeostasis and hibernation, which led him to the discovery of circannual rhythms in ground squirrels. Mrosovsky published three widely cited articles in JCPA (1980, 1983, 1985) characterizing circannual rhythms in two species of ground squirrels. After these initial forays into chronobiology, he delved into the study of circadian rhythms and specialized in how non-photic factors can influence circadian activity rhythms in the golden hamster. In his 1988 JCPA article, he showed that social interaction can phase-shift the circadian clock; in his 1989 JCPA article, he showed the same for running activity and that a surge in physical activity could explain the previously described phase-shifting effects of darkness pulses on hamster circadian rhythms. Mrosovsky is the author of four books (*Hibernation and the Hypothalamus*, Springer, 1971; *Conserving Sea Turtles*, British Herpetological Society, 1983; *Rheostasis: The Physiology of Change*, Oxford University Press, 1990; *Sustainable Use of Hawksbill Turtles: Contemporary Issues in Conservation*, Darwin: Key Centre for Tropical Wildlife Management, 2000) and author or co-author of more than 200 scientific articles, including 20 in JCPA.

**Brian Mulloney** is a well-known neuroethologist and Distinguished Emeritus Professor of neurobiology, physiology and behavior at the University of California Davis. He was educated at McGill University (BSc in zoology and chemistry in 1963), and at the University of California Berkeley (MA (1967) and PhD (1969) in zoology). Mulloney has dedicated his career to studying the crustacean central nervous system, and particularly its role in coordinating locomotion. He is particularly interested in how the coordinated action of crayfish swimmerets to produce power strokes during swimming are controlled neurally in each body segment, using electrophysiology and computational analyses to tease out the neural circuitry involved and to model its actions. Importantly, Mulloney discovered that each crayfish limb is controlled by its own neuronal module that functions independently of modules controlling other limbs. All these modules, in turn, are controlled by a separate circuit of coordinating interneurons that ensures coordinated locomotion. This work—which has beautifully revealed the neural basis of coordinated crustacean locomotion—led to a highly cited article on this topic in JCPA.

**Hanna Mustaparta** (born 1942, Harstad, Norway) is professor emerita, Chemosensory Lab, Norwegian University of Science and Technology. She received her master’s (1971; supervisor Kjell Døving) and *Dr. philos.* (1975) degrees from the University of Oslo. Her doctoral research was carried out at the Max Planck Institute for Behavioral Physiology in Seewiesen and the University of Gothenburg. After studying as a research fellow at Cornell University and Syracuse University, Mustaparta joined the faculty at the University of Odense and then moved to the University of Trondheim. She was professor of biology at the Norwegian University of Science and Technology in Trondheim from 1990 until retirement in 2012. Her 21 papers in JCPA analyze the selectivities and sensitivities of olfactory receptor and antennal lobe neurons in several species (heliothine moths, weevils) to biologically-relevant plant odors. This work contributes to the identification of molecular properties involved in the interaction between odorants and receptor neurons, and to the understanding of how tuning of different kinds of neurons contributes to odor identification.

**Werner Nachtigall** (1934-) was born in Saaz (Czech: Žatec), Czechoslovakia. He completed his studies in biology, physics, chemistry, and geography at the LMU Munich with a PhD, awarded in 1959. For his thesis research, carried out under the mentorship of Werner Jacobs at LMU Munich, he applied his training in technical physics and fluid mechanics to analyze how water beetles swim, dive, and steer in water, and how their body and their swimming organs are adapted to locomotion in fluid. This thesis research, published in JCPA (Nachtigall 1960), marked the beginning of a distinguished career in biomechanics and the then emerging discipline of bionics. After a two-year stint at the Institute of Radiation Biology in Neuherberg, he returned to the Zoological Institute of LMU Munich, where, in 1966, he received the *Habilitation* for his work on the biophysics of flight of insects and birds. In 1969, he was appointed to the position of *Ordinarius* and Director of the Zoological Institute of Saarland University, where he remained until his retirement in 2002. Of his more than three hundred publications, 21 papers appeared in JCPA.

**Toshiki Nagayama** (born 1956 in Japan) is professor emeritus at Yamagata University. He received his PhD in 1986 for research on the role of non-spiking interneurons in motor control of the crayfish at Hokkaido University under the direction of Mituhiko Hisada. Following a short postdoc with Hisada, he joined Malcolm Burrows from 1987–89 at the University of Cambridge, where he characterized the receptive fields of mechanosensory local interneurons in the metathoracic ganglion of the locust. In 1989, Nagayama returned to Hokkaido University, first as research associate and, in 1992, as lecturer pursuing research on the neural basis of escape, dominance, and agonistic behavior in crayfish and crickets. In 2007, he became full professor at Yamagata University until his retirement in 2021. In well over 70 publications, Nagayama contributed substantially to understanding the neural basis of social status and escape behavior in crayfish. Twenty-one of his publications on these topics appeared in JCPA.

**Gerbera Nalbach** (born 1960 in Bonn) studied biology and physics, first at the University of Bonn and then (1982) at the University of Tübingen. She passed the *Staatsexamen* (teacher state exam) in 1985 with research on the role of halteres in flies. She continued research on the functional role of the fly’s haltere system during her PhD thesis research at the Max Planck Institute for Biological Cybernetics in Tübingen, supervised by Roland Hengstenberg* and Karl Götz. Following her PhD in 1991, she joined the laboratories of Dezsö Varjú at the University of Tübingen (1992–93) and from 1993–94 the group of Gernot Wendler at the University of Cologne, continuing her studies on the role of halteres in dipteran flight control. Partly for health reasons, she left academia in 1994. Her six highly cited publications in JCPA focus on the flight control system of dipterans and on visual system organization in crabs.

**Peter M. Narins** (born 1943, Far Rockaway, New York) is professor emeritus of Integrative Biology & Physiology and of Ecology & Evolutionary Biology at the University of California Los Angeles. He earned his bachelor’s (1965) and master’s (1966) degrees in electrical engineering at Cornell University. Narins volunteered for the US Peace Corps from 1966–70, serving as an instructor in electrical engineering at Catholic University in Santiago, Chile. He returned to Cornell for his PhD in neurobiology and behavior (1976), under the mentorship of Robert R. Capranica*. There he examined neural and behavioral control of vocalizations in the Puerto Rican coqui frog, demonstrating sex differences in neural tuning in the inner ear. After postdoctoral work (1976–78) studying the mammalian auditory system with Edward F. Evans at the University of Keele, Narins accepted a faculty position at the University of California Los Angeles, where he remained until his retirement in 2022. Narins served as an associate editor of JCPA from 1995–2021, and as president of the International Society for Neuroethology from 2014–16. He is an elected Fellow of the Acoustical Society of America (1993), American Association for the Advancement of Science (1997), American Academy of Arts & Sciences (2022), Animal Behavior Society (1997), the Guggenheim Foundation (1993), and the International Society for Neuroethology (2016). He received the Award of Merit from the Association for Research in Otolaryngology (2019), and the Silver Medal in Animal Bioacoustics from the Acoustical Society of America (2021). He has traveled all over the world to conduct field work on acoustic communication in many species of frogs, and vibration sensitivity of golden moles and mole rats. His deep interest in auditory communication is also exemplified by his enthusiastic participation in the ham radio community; his contact record is now close to the maximum of 340. Narins’ 32 papers in JCPA focus on behavior and mechanisms of hearing and vocal communication in several species of anuran amphibians. These papers describe the spectral sensitivity of the frog’s inner ear and its relation to the frequency content of species-specific advertisement calls, the use of vibrometry to measure vibrations of the frog’s tympanum, the directionality of the frog’s inner ear, sensitivity and precision of evoked calling and selective phonotaxis in field tests, and the analysis (with Albert S. Feng*) of the ability of some frog species to hear ultrasound. In addition to his own papers, Narins edited, jointly with Daniel A. Llano, a Special Issue titled *Neuroethology of auditory systems: contributions in memory of Albert S. Feng* (Volume 209, Issue 1, 2023).

**Douglas M. Neil** (1947-) was born in Durham, UK. He received his BA honors degree in natural sciences (1969) and his PhD from the University of Cambridge (1972). From 1972–75, he was a research fellow at the Gatty Marine Laboratory, University of St Andrews. In 1975, Neil accepted a position in the Department of Zoology at the University of Glasgow, raising through the ranks to become professor of animal physiology (2007–11). He is currently honorary senior research fellow at the School of Cardiovascular & Metabolic Health, University of Glasgow. Neil served as president of the International Crustacean Council (2002–09). In 2005, he was the recipient of the Achievement Award conferred by The Crustacean Society, in recognition of his contributions to their international programs. His 12 papers in JCPA used large decapod crustaceans as model organisms to study sensory-motor integration, particularly in relation to equilibrium reactions of the postural and locomotory motor systems, and compensatory movements of the eyes. These studies combined a formal systems-based approach with neurophysiological analyses of the sensory, motor, and interneuron activities that underlie postural and locomotory behaviors. Work done in collaboration with François Clarac* analyzed, in palinurid lobsters, the transfer characteristics of sensory-motor reflex chains to uncover neuronal mechanisms by which postural command fiber systems are primed by sensory inputs.

**Robert M. Olberg** (1946-) was born in Evanston, Illinois. He is Florence B. Sherwood Professor of Life Sciences Emeritus at Union College. Olberg studied biology and received his BA from Rice University. He pursued graduate studies at the University of Washington and received his PhD supervised by John Palka in 1978 studying visual and multimodal interneurons in dragonflies. His fascination for insect brain neurons targeting flight motor circuits remained the focus of his research throughout his scientific career. For postdoctoral training, Olberg joined the group of Dietrich Schneider at the Max Planck Institute for Behavioral Physiology in Seewiesen. There, he discovered pheromone-triggered flip-flop neurons descending from the brain to thoracic ganglia in the silk moth. In 1981, he joined the Department of Biological Sciences at Union College as assistant professor, became associate professor in 1987, and full professor in 1996. There, he continued to analyze the transformation of sensory information into the control of behavior in insects. Most of his work is directed toward pray capture behavior in dragonflies. Olberg showed that a small group of visual interneurons in the dragonfly nerve cord that selectively respond to the movement of small objects are closely involved in directing prey-capture interception flights. Olberg published eight highly cited articles in JCPA on distance perception, prey selection and interception in dragonflies and the role of descending interneurons in these and other behaviors.

**Daniel Osorio** (1959-) is professor of neuroscience and head of the Department of Evolution, Behavior and Environment at the University of Sussex. He was educated at the University of Cambridge (BA and MA) and the Australian National University (PhD), where he studied with George Adrian Horridge*. He has been on the faculty at the University of Sussex since 1992. Osorio is a visual ecologist very well known for his fundamental studies of color perception in insects, birds and primates and for his work on camouflage in cephalopod mollusks. He also has a particular interest in evolution, especially the evolution of vision and visual signaling, and together with Misha Vorobyev* has revolutionized our understanding of the evolution of trichromatic color vision in primates. His most-cited paper in JCPA shows how colored oil droplets, UV vision and tetrachromacy in birds enhances their discrimination of plumage colors under natural illumination. In addition to his work on animal vision, Osorio is an advocate for the ethical treatment of animals during experimentation, and is active in creating guidelines for the use of cephalopods under the protection of the European Union animal welfare legislation.

**Julian Partridge** (1959-) is a marine biologist who was educated at the University of Bristol (BSc 1982 and PhD 1986), eventually becoming professor of zoology there in 2008. In 2014, he moved to the Biology Department at the University of Western Australia. Until his retirement in 2022, Partridge was director of the Oceans Institute and is now an adjunct senior research fellow at the same institute. He is also Fellow of the University of Western Australia Public Policy Institute. Partridge has had a long career studying the sensory systems of marine animals (particularly fish), and he has a particular interest for vision and eye design in the deep sea. During his career, particularly later as director of the Oceans Institute, Partridge actively promoted the translation of biological imaging systems to engineering. His current research projects span bio-inspired robotics, invertebrate vision, deep-sea sensory systems, the use of eDNA and underwater imaging for fish population assessment, and the development of insect meal as a feedstock for aquaculture. He has 11 well-cited papers in JCPA dealing with the visual pigments of fish and birds.

**Colin Stephenson Pittendrigh** (1918–1996; see obituary by Menaker 1996) is often regarded as the ‘father of the biological clock’. Together with Jürgen Aschoff and Erwin Bünning, he founded the field of modern chronobiology, which studies cyclic phenomena in living organisms. Pittendrigh was born in Witley Bay, on the coast of Northumberland, Britain and obtained a degree in botany from the University of Durham in 1940, now the University of Newcastle upon Tyne. During World War II, he was assigned to wartime service to try and improve the production of bananas and other fruit that was being shipped to the UK during the war. He also worked as a biologist for the Rockefeller Foundation and the government of Trinidad to control malaria near the military bases there. He studied the epidemiology of malaria transmitted by mosquitoes breeding in epiphytic bromeliad (“tanks” formed by overlapping leaves) in the forest canopy. He made acute observations on bromeliad distribution within forest canopies and between contrasting forest formations. He observed daily rhythms in mosquito activity patterns, particularly noting that peak activity times were different for different species at different canopy levels. His work with the biting rhythms of these mosquitoes was responsible for the development of his interest in biological rhythms, which later led to his experimental studies on eclosion rhythm in fruit flies. After the war, Pittendrigh attended Columbia University to study for his PhD in biology under the evolutionary geneticist Theodosius Dobzhansky. He received his PhD from Columbia University in 1947 and then joined the faculty at Princeton University as an assistant professor of biology, where he started his chronobiology research and where he trained Michael Menaker*. While at Princeton, he gained his U.S. citizenship in 1950 and served as dean of graduate studies from 1965–69. In 1969, he joined the faculty at Stanford University where he founded the Human Biology program and later became the director of the Hopkins Marine Station. Pittendrigh retired from Stanford in 1984 and moved to Bozeman, Montana. Here, he continued his studies of biological clocks, working with the faculty and lecturing at Montana State University Bozeman. Pittendrigh is known for his careful descriptions of the properties of the circadian clock in fruit flies, rodents, and other species, and providing the first formal models of how circadian rhythms entrain (synchronize) to local light–dark cycles. He published seven papers in JCPA of which the series of five papers with Serge Daan* in 1976 (‘A functional analysis of circadian pacemakers in nocturnal rodents’, Parts I-V) are among his most influential ones. Pittendrigh’s research continues to have an impact on the field of chronobiology even after his death. The Society for Research on Biological Rhythms holds biennial lectures named in honor of Pittendrigh and Aschoff.

**Roy E. Ritzmann** (1947-) was born in Chicago. He graduated from the University of Iowa in zoology in 1969 and received a PhD in biology from the University of Virginia in 1974. During three years of postdoctoral training in the laboratory of Jeffrey M. Camhi* at Cornell University, he began working with insects on the neural circuitry underlying escape systems. He then joined the faculty of Case Western Reserve University, where he subsequently progressed through the ranks to full professor. Ritzmann’s research has aimed at determining how animals move through complex terrain in their natural habitat. To address this question, he uses mainly cockroaches because, while they are very agile animals, they are also well suited for correlating their body and leg movements (recorded using high-speed video systems) with electrical activity (recorded from neurons and muscles). In collaboration with engineers, Ritzmann has also used data generated through this work to build biologically inspired robots. In turn, these robots serve as hardware models for gaining insight into the control of locomotion in animals. His 20 publications in JCPA have attracted over 1100 citations.

**Gene E. Robinson** (1955-) pioneered the application of genomics to the study of social behavior and led the effort to sequence the honeybee genome. He received his bachelor’s in life sciences (1977), master’s in entomology (1982), and PhD in entomology (1986) from Cornell University. From 1986–89, he joined Robert E. Page at Ohio State University for postdoctoral studies on the genetic structure and division of labor in honeybee societies. In 1989, he accepted the offer for an assistant professorship at the Department of Entomology of the University of Illinois Urbana-Champaign, where he was promoted to associate professor in 1994 and to full professor in 1997. Robinson served as Director of the Neuroscience Program from 2001–11, as William Arends Professor of Integrative Biology from 2003–8, as IGB Theme Leader and Interim Director from 2004–11 and 2011–12, respectively, and as Interim Dean of the College of Liberal Arts & Sciences from 2020–21. Currently, he is the director of the Carl R. Woese Institute for Genomic Biology and director of the Bee Research Facility at the University of Illinois. In addition, he holds a University Swanlund Chair and a Center for Advanced Study Professorship. He has received numerous awards and honors including honorary doctorates. Among these are Fellow of the American Association for the Advancement of Science (1996), Fellow and Founders Memorial Award (Entomological Society of America, 2009), Fellow and Distinguished Behaviorist (Animal Behavior Society, 2006), Distinguished Scientist Award (International Behavioral Genetics Society), Guggenheim Fellowship (2006), Fulbright Fellowship (1995–96), NIH Pioneer Award (2019), and the Wolf Prize in Agriculture (2018). Robinson is a member of the American Academy of Arts & Sciences (2004), US National Academy of Sciences (2005), US National Academy of Medicine (2018), and the American Philosophical Society (2021). Authoring or co-authoring almost 350 publications, Robinson has made fundamental advances in understanding the endocrine, neural, and genetic regulation of behavior at the individual and colony levels in honeybees. His discoveries have significantly advanced the understanding of the role of genes, hormones, and neurochemicals in the mechanisms and evolution of social behavior. In his 15 papers in JCPA, Robinson describes the effects of biogenic amines and different hormones on the division of labor in honeybees as well as on caste determination in bumblebees.

**Heinrich Römer** (born 1948 in Kindel, Germany) is professor emeritus at Karl-Franzens University of Graz. Römer studied biology at the University of Marburg and Ruhr University Bochum, where he joined the group of Johann Schwartzkopff. He received his doctorate (*Dr. rer. nat.*) in 1975, analyzing the functional characteristics of auditory fibers in locusts. Following positions as research assistant and lecturer at the University of Stuttgart and Ruhr University Bochum, he received his *Habilitation* from Ruhr University Bochum in 1986. In 1992, he became professor for zoology at the University of Graz. Throughout his scientific career, Römer studied acoustic communication and processing in insects, both in the field and in the laboratory, focusing on grasshoppers and bush crickets. Field trips to Australia, South Africa, and Panama revealed the importance of the ecological situation on the evolution of acoustic signaling. As illustrated in 32 publications in JCPA, Römer paid particular attention to the sensory ecology of the species under study and consequently developed methods to record from single auditory neurons in the natural habitat of the animal under investigation.

**Bernhard Ronacher** (born 1949 in Innsbruck) studied biology at the Universities of Innsbruck and Freiburg. Supervised by Bernhard Hassenstein, he received his doctorate (*Dr. rer. nat*.) in 1974 from the University of Freiburg working on pattern recognition in honeybees. He stayed at the University of Freiburg until 1979, then joined the group of Otto von Helversen at the University of Erlangen as research assistant and senior research assistant. Ronacher received his *Habilitation* in 1986, and became professor of animal physiology at Humboldt University Berlin in 1994. Since 2007, he is a member of the *Akademie der Wissenschaften zu Göttingen* (Göttingen Academy of Sciences and Humanities) and since 2009 member of the German National Academy of Sciences (Leopoldina). The focus of Ronacher’s research is on three neuroethological topics: pattern recognition in honeybees, song production and recognition in grasshoppers, and, more recently, spatial orientation in ants. Hallmarks of his research are sophisticated experimental designs and a particular focus on ecophysiological aspects of signaling and behavior. In JCPA, he published 42 articles on spatial orientation of desert ants and acoustic communication and courtship behavior in grasshoppers.

**Gary J. Rose** (1954-) was born in San Diego, California. After graduating from the University of California San Diego in biology in 1977, he enrolled into the neurobiology and behavior graduate program at Cornell University. There, in the laboratory of Robert R. Capranica*, he became interested in the question that has remained central to his research ever since— how does the brain process information encoded in acoustic signals relevant to the natural behavior of animals? In his thesis research, he addressed this question by examining the response of the auditory system to modulated sound in anuran amphibians. After receiving his PhD in 1983, he joined the laboratory of Walter Heiligenberg* at the Scripps Institution of Oceanography of the University of California San Diego for postdoctoral training. There, a major focus of his research was the neurophysiological characterization of central structures that serve as neural filters of temporal information encoded in electric discharges of electric fish. In 1988, Rose was appointed to an assistant professorship at the University of Utah, where he subsequently progressed through the faculty ranks to full professor. His main research interest has remained the study of neural mechanisms of audition in anurans, which he explores by combining behavioral investigations in the field and laboratory with neurophysiological analysis of single neurons and neural circuits. Rose’s first paper in JCPA appeared in 1984. Since then, he has published another 18 articles in the Journal, placing him in the league of the most prolific authors of JCPA over the last 100 years.

**Olav Sand** (born 1946 in Vefsn, Norway) studied biology and chemistry at the University of Oslo, from which he also received his PhD in 1973. Following appointments as a research fellow, he commenced his faculty career as an associate professor at the Norwegian College of Veterinary Medicine in 1978. In 1982, he joined the University of Oslo, with which he remained affiliated throughout his tenure as associate and full professor. Upon his retirement, he became an emeritus professor in 2014. Although Sand held all his appointments at Norwegian institutions throughout his career, he collaborated intensively with research labs abroad, many of which he visited for extended periods of time. The countries in which he worked as a visiting scholar included Scotland, the United States, Japan, Denmark, England, Italy, Australia, Belgium, and Ireland. Sand’s research interests span a broad range of themes, including signal transduction in endocrine cells, membrane-permeabilizing effects of bacterial peptides, physiology and behavior of ciliates, as well as hearing and lateral line physiology in fish. The latter interest is reflected by the six highly cited papers that he published in JCPA.

**David C. Sandeman** (1936-) was born in Springs, South Africa. He received his BSc and MSc from the University of Natal, South Africa. For his PhD he moved to the University of St. Andrews. Under the supervision of George Adrian Horridge* he investigated the neural control of eye movements in the crab and received his PhD in 1964. Following postdoctoral training in the laboratory of Theodore H. Bullock*, he became lecturer at the University of St. Andrews until 1969 when he moved with Horridge* to the Research School of Biology at the Australian National University. In 1982, he became professor of zoology at the University of South New Wales. In addition to continuing studies on eye movements, in particular vestibulo-ocular reflexes and their neural control in crabs, Sandeman’s topics of interest broadened into analyses of the antennal and olfactory system, general brain organization in decapod crustacea, serotonin function in the crayfish brain, as well as the neural underpinnings of the balancing function of halteres in flies. As a visiting professor in Würzburg (2001–03) he collaborated with Jürgen Tautz on honeybee recruitment in the bee hive and more recently, as a research scholar at Wellesley College with Barbara Beltz (2002–09) on neural development and neurogenesis in the decapod brain. Among his many honors, Sandeman has been Fellow of the *Wissenschaftskolleg* (Institute for Advanced Study) in Berlin and is member of the *Akademie der Wissenschaften und der Literatur* (Academy of Sciences and Literature) in Mainz. The full breadth of his research is impressively represented by 21 articles in JCPA ranging from behavioral studies on antennal and eye movements in crustacea, honeybee recruitment, olfactory coding in crayfish, to the role of halteres in flies.

**Henning Scheich** (1942-) was born in Wuppertal, Germany, and studied medicine and philosophy at the Universities of Cologne, Munich, and Montpellier. He completed his medical studies with the state examination at LMU Munich in 1966. From 1967–69, Scheich conducted PhD research on the human electroencephalogram at the Max Planck Institute of Psychiatry, Munich, in the department of Otto Creutzfeldt. From 1969–72, he worked in the laboratory of Theodore H. Bullock*, University of California San Diego, investigating the jamming avoidance response of electric fish and its neurophysiological basis. This research was published in JCPA in 1972. In 1972, Scheich started his own research group at the Max Planck Institute for Biophysical Chemistry, Göttingen. In 1974, he accepted an appointment at the Technical University of Darmstadt to a professorship in zoology and neurobiology. He continued to work on the neurobiology of acoustic communication and undertook research trips to the Amazon basin, Central Africa, Thailand, Puerto Rico, and Australia to study the communication behavior in birds, electric fish, and other vertebrates. He discovered infrasonic hearing in birds and the electric sense in platypus. In 1982, Scheich became director and department head of the Leibniz Institute for Neurobiology, Magdeburg, and from 1994 onward he was additionally professor of physiology at the Medical Faculty of Otto von Guericke University Magdeburg. Scheich's research activities at the Leibniz Institute for Neurobiology focus on the organization of auditory and vocalization behavior in animals and humans and the role of the auditory cortex in learning processes. Since 2010, he leads the emeritus group ‘Life-Long Learning.’ Of the 242 articles authored or coauthored, Scheich published 17 original research articles in JCPA. He had a formative influence on the research landscape in Germany and was awarded several honors for his commitment to science, scientific self-governance, and policy advice.

**Klaus Schildberger** (1951-) was born in Berlin. He studied biology at the Free University of Berlin and received his *Diplom* (master’s) in 1978 with a thesis on visual learning in harnessed honeybees, supervised by Joachim Erber*. He joined the group of Friedrich-Wilhelm Schürmann in Göttingen for a dissertation on the physiology and morphology of interneurons in the brain of crickets and received his *Dr. rer. nat.* in 1982. Crickets remained the subject of his scientific interest. As research associate in the group of Franz Huber* at the Max-Planck Institute for Behavioral Physiology, he focused on analyzing the neural processing of auditory signals in the brain of crickets and proposed a model circuit for auditory recognition of the male calling song in the cricket brain. Following his *Habilitation* from LMU Munich, he became professor of general zoology at the University of Leipzig in 1994. There his interest shifted to behavioral studies on male-male aggression in crickets and the role of neuromodulators such as octopamine, serotonin, and nitric oxide in modulating agonistic male-male encounters. Schildberger published eight well-cited articles on cricket phonotaxis and the neural basis of cricket acoustic communication in JCPA. He retired in 2016.

**Hans-Ulrich Schnitzler** (born 1939, Bad Urach, Germany) is senior professor in the Institute of Neurobiology at the University of Tübingen. After service in the German Navy, he pursued doctoral studies at the University of Tübingen (PhD 1968) under the direction of Franz Peter Möhres. In the course of his work, he discovered the phenomenon of Doppler shift compensation in constant frequency horseshoe bats. Schnitzler conducted postdoctoral research at Rockefeller University (1968–69) where he continued his analyses of constant-frequency echolocation and Doppler shift compensation with Donald R. Griffin. He returned to the University of Tübingen in 1969, where he conducted electrophysiological experiments on constant-frequency echolocating bats with Gerhard Neuweiler and Gerd Schuller. Schnitzler completed his *Habilitation* at the University of Tübingen in 1973, and then accepted faculty positions first at the University of Frankfurt (1973–76) and then at the University of Marburg (1976–80). He returned to the University of Tübingen in 1980 as professor and chair of Animal Physiology and remained there until his retirement in 2008, when he was named the University’s first senior professor. He is a founding member and Fellow (2020) of the International Society for Neuroethology. Schnitzler’s research combines laboratory behavioral experiments and acoustic measurements with field observations and recordings of echolocating bats in natural foraging contexts. His field work, which he continues to the present, on diverse bat species has taken him across the globe. On the basis of this research, he developed a classification scheme in which different bat species are placed into guilds based on their local ecologies, foraging behaviors, and echolocation calls. His 27 papers in JCPA describe how diverse species of echolocating bats detect and process echoes in cluttered environments, how constant-frequency bats use Doppler shift compensation to detect fluttering insects, and how bats control flight and vocal output in narrow flyways.

**Allen I. Selverston** (1936-) was born in Chicago. He earned his bachelor’s degree in physiology at the University of California Berkeley in 1962 and received his PhD in neurophysiology under the mentorship of Graham Hoyle at the University of Oregon in 1967. From 1967–69, he pursued postdoctoral work in the lab of Donald Kennedy at Stanford University. In 1969, he joined as assistant professor the University of California San Diego, where he progressed through the faculty ranks to full professor. After a three-year stint as director of the Institute of Neurobiology at the University of Puerto Rico from 1997–2000, he returned to the University of California San Diego as research professor. Since 2016, he has been emeritus professor. Selverston is best known for his morphological, physiological, and computational studies of the stomatogastric ganglion of decapod crustaceans, whose approximately 30 neurons produce two distinct rhythmic motor patterns. His 11 papers published in JCPA reflect this work and have contributed significantly to our understanding of how oscillatory behavior is generated by central pattern generators consisting of small neural circuits. For his scientific achievements, Selverston received several notable distinctions, including a Guggenheim Fellowship and a Humboldt Senior Scientist Award.

**Andrea Megela Simmons** (1951-) was born in McKeesport, Pennsylvania. She earned her bachelor of arts degree at the University of Pennsylvania, where she studied history and psychology. It was there, influenced by Norman T. Adler, that she became interested in biological psychology. During her doctoral studies in psychology at Harvard University, she continued to pursue training in psychobiology, with a focus on auditory physiology and perception. After receiving her PhD in 1978, she was formally introduced to neuroethology while working as a postdoctoral researcher in the laboratory of Robert R. Capranica* at Cornell University. Her neuroethological training also benefited from a semester-long stint as a visiting researcher with Jörg-Peter Ewert* at the University of Kassel. In 1982, Simmons joined the faculty of Brown University, where she currently holds appointments as full professor in the Department of Cognitive, Linguistic & Psychological Sciences and the Department of Neuroscience. She is a Fellow of the Acoustical Society of America (elected 2001). She is best known for her neuroethological studies of acoustic communication and auditory perception, primarily in adult and developing anuran amphibians, using behavioral, neurophysiological, and immunohistochemical methods. In recent years, she has expanded her research portfolio to include behavioral and electrophysiological studies of echolocation in bats. Out of over one hundred publications, 20 have appeared in JCPA. Simmons also serves on the Editorial Board of the Journal.

**Allan Snyder** (1942-) was educated at Pennsylvania State University (BSc in electrical engineering 1963), Harvard University and MIT (master’s degrees in science 1965 and 1967) and University College London (PhD 1969, and DSc). He is director for the Centre of the Mind at the University of Sydney and holds its 150th Anniversary Chair of Science and the Mind. He also holds the Peter Karmel Chair of Science and the Mind at the Australian National University. He is the recipient of many prestigious awards and fellowships, including election as Fellow of the Australian Academy of Science (1985), Fellow of the Australian Academy of Technology and Engineering (1987) and Fellow of the Royal Society of London (1990). He won the Harrie Massey Medal and Award in 1996, the Australia Prize in 1997, the Marconi Prize in 2001, and the Clifford Patterson Prize in 2001. Snyder is an optical physicist and in his early career made seminal contributions to our understanding of the optical functions of eyes, both vertebrate and invertebrate. He mathematically formalized the optics of compound eyes and their capacity to code information, major intellectual contributions that were published in JCPA. Snyder also made significant contributions to our understanding of photoreceptor optics, particularly their waveguiding properties. His 1983 book *Optical Waveguide Theory* (together with John D. Love; Springer Verlag) is now a classic. His later work turned towards understanding higher cognitive function in humans, with a special interest in savant autism and the neural basis of the ability of savants to recall minute details and to perform lightening quick calculations.

**Mandyam Veerambudi Srinivasan** (1948-) is emeritus professor at the Queensland Brain Institute at the University of Queensland. He was educated at Bangalore University (bachelor's degree in electrical engineering 1967), the Indian Institute of Science Bangalore (master’s degree 1970), and Yale University (MPhil 1973 and PhD 1977). He received a DSc in neuroethology from the Australian National University in 1994, working with George Adrian Horridge*. He is the recipient of many prestigious awards and fellowships, including election as Fellow of the Australian Academy of Science (1995), Fellow of the Royal Society of London (2001), an inaugural Federation Fellowship (2001), the Australasian Science Prize (2001), the Australian Centenary Medal (2003), Prime Minister’s Prize for Science (2006), Rank Prize for Optoelectronics (2008), and Membership of the Order of Australia (2012). Srinivasan is renowned for his remarkable discoveries in the field of insect vision, particularly concerning their visual control of flight and navigation, with many of these discoveries being published in JCPA (in 23 well cited papers). Using honeybees as a model animal, Srinivasan has shown that during flight insects measure optic flow and attempt to hold it constant in both eyes to measure distance travelled (which is signaled to other bees through the waggle dance), to reduce (and finally zero) velocity to facilitate a smooth landing, and to fly between and around obstacles to avoid collisions. The principles he has gleaned from his studies on bees have had significant impact in robotics, particularly for the creation of simple and efficient control systems for autonomously flying vehicles. In more recent times, he has also revealed similar principles in birds.

**Doekele Stavenga** (1942-) is an optical physicist and emeritus professor and former head of the Department of Physics at the University of Groningen, where he also received his PhD in 1974. Stavenga has had a long and distinguished career specializing in the visual optics of insect eyes, particularly in the neural superposition eyes of flies. He was the first to measure the refractive index of an insect photoreceptor and has made seminal contributions to our understanding of insect pupil mechanisms and the function of insect visual pigments. Stavenga was also the first to formalize the effects of waveguide modes on the spatial and spectral properties of insect photoreceptors, and to quantify the effects of the moveable pigment pupil on these properties. During two highly productive years in the laboratories of George Adrian Horridge* and Allan Snyder* at the Australian National University in Canberra (1975–1977), Stavenga collaborated with Snyder* and Simon Laughlin* to formalize the physical basis of information uptake in insect compound eyes. Together with his students Hans van Hateren and Robert de Ruyter van Steveninck, this work led to watersheds in our understanding of how eyes maximize information acquisition from natural scenes at different light levels. Stavenga’s more recent work has focused on the physics and functions of structural coloration in insects, flowers and birds. A large fraction of his work (46 articles) is published in JCPA, and many of these papers are now classics in the field.

**Stephan Steinlechner** (1950-) was born in Pullach near Munich. He studied biology at the Technical University Munich, LMU Munich, and Goethe University Frankfurt. He was introduced to chronobiology while he did his diploma thesis in the laboratory of Jürgen Aschoff at the Max Planck Institute for Behavioral Physiology in Erling-Andechs. In 1981, he received a PhD for his thesis research, conducted under the supervision of Gerhard Heldmaier, on seasonal thermogenetic adaptation in the Djungarian hamster. Subsequently, as a postdoctoral fellow, he joined the laboratory of Russell J. Reiter at the University of Texas San Antonio for one year to study the control of serotonin/melatonin levels in the pineal gland of various rodents. After a stint as a visiting professor at Juniata College, he was appointed to a full professorship at the University of Veterinary Medicine Hannover in 1992, where he remained until his retirement in 2014. Currently, he holds the status of senior researcher. Steinlechner contributed significantly to our understanding of the mechanisms of seasonal adaptation in small mammals. In his six highly cited articles published in JCPA, he reported that the changing photoperiod, but not the decreasing temperature, is responsible for the seasonal adaptations of hamsters, and that melatonin acts as an endocrine signal for night length. He also showed that physical activity influences the photoperiodic responses and the reproductive success of hamsters. Furthermore, he demonstrated that the Djungarian hamster most likely has a labile circadian clock that can be disturbed by light pulses during the night.

**Gunther S. Stent** (1924–2008; for an obituary see Barondes 2011) was a distinguished scholar who made important contributions not only to molecular biology and neurobiology but also to philosophy and the training of generations of young scientists. Born Günter Siegmund Stensch into an affluent Jewish family in Berlin, he was forced to flee Germany after the *Kristallnacht* in 1938. Two years later, he came to the United States, where he changed his family name and anglicized his first name. Stent received his BS and PhD in physical chemistry from the University of Illinois. In 1948, he joined, as a postdoctoral researcher, the famous ‘phage group’ around Max Delbrück at the California Institute of Technology, who became not only his academic mentor but also a life-long father figure. In 1952, Stent took a faculty position at the University of California Berkeley, where he remained until shortly before his death. Stent made important contributions to the study of DNA replication, as well as RNA transcription and translation. In the late 1960s, his interests shifted to neurobiology, with a major focus on neural circuits of central pattern generators that control rhythmic behavior in leeches. Subsequently, his interests shifted again, now to the study of neuronal migration, axonal arborization, and cell lineage in the developing nervous system of leeches. Finally, he underwent another major transformation by turning his attention to philosophical topics, such as the nature of free will and consciousness. Besides his own research, his legacy is the impact he had on generations of young scientists by establishing a school of highly gifted and successful students, creating new departments and academic programs at the University of California Berkeley, and writing extremely popular textbooks. In JCPA, he published seven highly cited papers on neural control of heartbeat and swimming in leeches.

**Nicholas J. Strausfeld** (born in 1942 in Brighton, UK) is a neuroscientist with a fascination for arthropod nervous systems. He studied biology at University College London, where he received his PhD in 1968 under the supervision of David Blest. Together they refined classical silver impregnation and Golgi staining techniques for application to insect brains, specifically the optic lobe of the privet hawkmoth. Fascinated by the staggering beauty of the stained neurons, Strausfeld developed a long-standing interest in the anatomical organization of insect brains. During research positions at Goethe University Frankfurt, the University of Tübingen, and a group leader position (1975–86) at the European Molecular Biology Laboratory, Heidelberg, he studied the cellular organization of insect—specifically fly—visual systems. He completed his *Habilitation* at Goethe University Frankfurt in 1985. The monumental books *Atlas of an Insect Brain* (Springer, 1975) and *Arthropod Brains: Evolution, Functional Elegance, and Historical Significance* (Harvard University Press, 2012) established him as a world leading neuroanatomist. In 1997, Strausfeld accepted a position as professor of neuroscience at the newly founded Arizona Research Laboratories, Division of Neurobiology and served for many years as director of the Center for Insect Science at the University of Arizona. He received the Alexander v. Humboldt Senior Research Prize (2001) and is a Fellow of the Royal Society of London (2002). Strausfeld and his trainees analyzed the role of interneurons in various parts of the insect nervous system. Comparative studies not only in insects, but also in spiders, various crustaceans and other arthropods led to an increasing interest in questions of brain evolution in arthropods, including studies of fossils. Among well over 180 publications, he published 12 highly cited papers on fly motion vision and the fly neck motor system in JCPA.

**Marianne Vater** (1952-) was born in Rüdesheim, Germany. She studied biology at Goethe University Frankfurt and received her doctorate (*Dr. rer. nat.*) in 1979 for research on the auditory physiology in echolocating bats, supervised by Gerhard Neuweiler. She stayed in Neuweiler’s group for postdoctoral training until 1981, characterizing neurons in the inferior colliculus of horseshoe bats. In 1981, she followed Neuweiler as *Akademische Rätin* (equivalent to lecturer) to LMU Munich, where she received her *Habilitation* in 1987. Following an interim professorship at the Department of Zoology at the University of Regensburg and a Heisenberg stipend, she was appointed as full professor of general zoology at the University of Potsdam in 1996 and retired in 2018. Throughout her career, Vater’s focus of interest was the neurobiology of the auditory system of echolocating bats. In addition to electrophysiological studies on auditory neurons at various stages of the auditory system (done in part in collaboration with Albert S. Feng*), she analyzed the development of echolocation and the functional and comparative cochlear anatomy of bats. More recently, she also contributed to studies on the ontogeny of electric organ discharges in weakly electric fish. Vater published 19 articles in JCPA reflecting the full spectrum of her scientific achievements.

**Dora Selma Fix Ventura** (1939-) graduated in psychology from the Faculty of Philosophy and Sciences of the University of São Paulo (1961), and completed her master's and doctorate in experimental psychology at Columbia University (MA, 1964; PhD, 1968). She became a full professor at the Institute of Psychology of the University of São Paulo in 1990. There she had already founded the Laboratory for Sensory Psychophysiology in 1968 to investigate the neuronal mechanisms of vision using behavioral and electrophysiological methods. After her appointment as a full professor, she also founded the Vision Laboratory, which is dedicated to applied research in the field of psychophysics and clinical visual electrophysiology in order to investigate neurodegenerative diseases of the visual system that are of genetic or metabolic origin or are due to the effects of neurotoxic substances. She has published internationally in both areas. Ventura was president and vice president of several national and international scientific organizations and was awarded the National Order of Scientific Merit, the CAPES 50 Years Medal for 50 years and the Brazilian Medal of Neuroscience. In her 12 articles in JCPA, Ventura characterized the spectral sensitivity of different compound eyes of insects, the physiological response properties of photoreceptor and lamina monopolar cells of Hymenoptera, and their visual sensitivity and adaptation. Some of this work is in collaboration with John Manuel de Souza*. In her later work, she also investigated color vision in goldfish and capuchin monkeys.

**Karl von Frisch** (1886–1982) was born into a family of distinguished medical doctors, scholars, and professors in Vienna. He first enrolled in medicine at the University of Vienna, but after five semesters transferred to the LMU Munich to read zoology under Richard Hertwig. For both his PhD (at the Institute for Experimental Biology in Vienna) and *Habilitation* (at LMU Munich), he studied color changes in fish. He also worked as a scientific assistant of Hertwig, but his tenure was disrupted by World War I, during which he served by performing bacteriological and medical work at a Red Cross hospital in Vienna. In 1921, he accepted the offer to become *Ordinarius* (full professor) and director of the Zoological Institute at the University of Rostock, but just two years later he moved to a similar position at the University of Breslau. After another two years, he succeeded his mentor Richard Hertwig at LMU Munich. Under von Frisch’s leadership and funded by a major grant from the Rockefeller Foundation, in 1931–32 the former Zoological Institute was replaced by what was then the most modern and best-equipped institute for zoological research in Europe. When most of the institute building was destroyed toward the end of World War II, he resigned from his position in 1946 to assume a professorship at the University of Graz, yet he returned to LMU Munich in 1950. He became emeritus professor in 1958. Among the numerous accolades he received are the Order of Merit for Sciences and Arts (1952), the Kalinga Prize (1959), the Austrian Medal of Honor for Science and Art (1960), the Balzan Prize for Biology (1963), the Nobel Prize for Medicine or Physiology (1973), the Distinguished Service Cross with Star and Ribbon of the Order of Merit of the German Federal Republic (1974), as well as honorary doctorates from the University of Bern (1949), the Technical University of Zurich (1955), the University of Graz (1957), Harvard University (1963), the University of Tübingen (1964), and the University of Rostock (1969). He was also a fellow or honorary member of many academic and scientific societies. Von Frisch pioneered research at the interface of sensory physiology and ethology. His many achievements include the discoveries of color vision in fish and honeybees, hearing in fish, *Schreckstoff* (alarm substance) and its biological function in fish, dance language in bees, and polarization vision and its role in orientation in bees. Besides his scientific publications, von Frisch was also highly successful in contributing through several popular books to the public understanding of science. Although jointly with Alfred Kühn, he had founded the predecessor of JCPA, and served as its editor-in-chief for 36 years, he published only 9 articles in the Journal. Yet, they were highly impactful, as indicated by a mean citation rate of nearly 100.

**Misha Mikhail Vorobyev** is a senior lecturer in optometry and vision science at the University of Auckland. Vorobyev was educated at Leningrad University (diploma in physics 1980) and the USSR Academy of Sciences (PhD in physics and mathematics 1989). Vorobyev has made paradigm-shifting contributions to our understanding of color vision in insects, birds and mammals, and in particular to our understanding of its physiological underpinnings and its evolution (with many highly cited publications in JCPA). Using mathematical modelling he showed that physiological noise present in the different spectral classes of photoreceptors provides the ultimate limit to the perception and discrimination of color (the highly influential ‘noise-limited model of color vision’). Together with Daniel Osorio*, Vorobyev also revolutionized our understanding of the evolution of trichromatic color vision in primates. By using modelling, he showed that the particular form of color vision found in primates likely evolved for distinguishing ripe from unripe fruit. In more recent years he has turned his attentions to polarization vision in cephalopods.

**Eric J. Warrant** (1962-), born in Gosford, New South Wales, is professor of functional zoology at Lund University. He received his bachelor’s degree in physics (1985) at the University of New South Wales and his PhD (1990) in neurobiology at Australian National University. His dissertation research, conducted under the mentorship of George Adrian Horridge* and Peter McIntyre, modeled and experimentally probed the optics of the superposition eyes of dung beetles. Warrant conducted postdoctoral research with Dan Nilsson in the Department of Zoology at Lund (1990), working on optics of insect eyes. He then stayed in Lund as a faculty member, and was appointed professor in 2002. He is a founding member and head of the Lund Vision Group, a leading research group for comparative vision research. His former postdoctoral student, Almut Kelber*, is also one of the Top 100 Authors. Warrant was appointed Schering Fellow (1997–98) of the Institute of Advanced Studies in Berlin. He is a Fellow of the Royal Physiographic Society of Lund (2005), a foreign Fellow of the Royal Danish Academy of Sciences and Letters (2008), Fellow of Royal Institute of Navigation (2019) and a member of the German National Academy of Sciences (Leopoldina, 2023). He served as president of the International Society for Neuroethology from 2018–22, and is currently an associate editor of JCPA. Warrant’s research examines visual optics and visual navigation, with a focus on understanding how animals see in dim light. His 24 papers in JCPA, including five from his doctoral dissertation, model and analyze the operation of visual systems of a wide range of species, including dung beetles, bony fishes, elasmobranchs, carpenter bees, seals, and spiders. He has published a short memoir in this Special Issue (Warrant 2023).

**Rüdiger Wehner** (born 1940 in Nürnberg, Germany) is emeritus professor of zoology at the University of Zurich. Wehner studied biology, chemistry and philosophy at the University of Frankfurt. He obtained his doctorate (*Dr. rer.nat*.) supervised by Martin Lindauer* in 1967 and received his *Habilitation* at the University of Zurich working with Ernst Hadorn. Following a research stay at Yale University, he accepted an offer for professor of zoology at University of Zurich in 1974 and served as director of the Zoological Institute for many years. Wehner is a member of the German National Academy of Sciences (Leopoldina,1985) and of the Academia Europaea (1989). He is a recipient of the Karl Ritter von Frisch Medal and Science Prize of the German Zoological Society (1994), the Carus Medal of the German Academy of Sciences (1993), King Faisal International Prize for Science (2008), and the University Silver Medal of the University of Tübingen (2009). He is a Fellow of the International Society for Neuroethology (2012), and has received several honorary doctorates. Starting from his doctoral thesis, Wehner’s scientific interest is focused on the biology of social insects, in particular spatial orientation in honeybees and desert ants. His research covering several decades of field and laboratory work established the desert ant as a model organism in research on spatial orientation and provided general insights into mechanisms of sky compass orientation, magnetic orientation and path integration. His broad scientific interest is perhaps best illustrated by his role as author of several editions of the German textbook *Zoologie* (Zoology; together with Walter Gehring; Georg Thieme Verlag). In JCPA, he published 42 widely cited papers on navigation and its underlying mechanisms in honeybees and desert ants.

**Roswitha Wiltschko** (1947-) was born in Bielefeld, Germany. She studied zoology, botany, and paleontology at Goethe University Frankfurt, from which she also received her PhD (in 1979) and her *Habilitation* (in 1990). She has remained affiliated with the University of Frankfurt throughout her subsequent scientific career, including her appointment to an adjunct professorship in 2006. For most of her research, she has collaborated with her spouse, Wolfgang Wiltschko*. Together, they have studied mechanisms that provide avian species, including migratory birds and homing pigeons, with navigational information. In 2009, both became honorary fellows of the Royal Institute of Navigation in recognition of their research. They jointly have authored *Magnetic Orientation in Animals* (Springer-Verlag, 1998), a must-read book for anyone interested in animal navigation and orientation. Most of the 19 papers that Wiltschko has (co-)authored in JCPA have focused on the role of magnetoreception in the orientation of birds, with particular emphasis in recent years on the physiology of the two types of putative magnetoreceptors, based on magnetite and the avian cryptochrome 1a, respectively.

**Wolfgang Wiltschko** (1938-) was born in Kienberg, Germany. He studied zoology, botany, and chemistry at Goethe University Frankfurt, where he remained, apart from short research visits to other academic institutions, throughout his scientific career. In his thesis research, carried out in the laboratory of Friedrich Wilhelm Merkel on European robins, he succeeded in obtaining the first experimental evidence that migratory birds can use magnetic cues for orientation. After earning his PhD in 1967, he worked as a scientific assistant at the Zoological Institute. In 1972, he received the *Habilitation*. In 1974, he joined the laboratory of William T. Keeton* at Cornell University, where he studied the role of magnetic compass in homing. From 1975–2003, he was a professor of zoology at Goethe University Frankfurt, where he continued this line of research. His research efforts, frequently joined by his spouse Roswitha Wiltschko*, focused on various aspects of the behavior of migratory birds, including the role of the magnetic compass in the establishment of magnetic maps, the impact of infrasound and olfactory information on orientation and navigation, and physiological mechanisms underlying magnetic compass orientation. Even after retirement, he continued his research. In JCPA, Wiltschko published 19 highly cited papers, which are testament to his reputation as one of the leading pioneers in the study of the magnetic sense in animals.

**Reinhard Wolf** (1947-) was born in Wetzlar, Germany. He studied physical engineering at the Heilbronn University of Applied Sciences, where he graduated in 1975. When he came across the work of Werner Reichardt, Karl Götz, and Martin Heisenberg* at the Max Planck Institute for Cybernetics, Tübingen, Wolf was fascinated by the idea of using engineering methods, combined with the use of brain mutants to understand the function of the ‘relatively simple’ insect brain. He accepted a position that Heisenberg offered him at the newly founded Chair of Genetics and Microbiology (later Chair of Genetics and Neurobiology) at the University of Würzburg in 1976. However, after Wolf and Heisenberg had realized that the fly’s behavior could not be explained by simple cybernetic approaches, they focused their interest on the spontaneous behavior generated by the fly brain itself. For more than 40 years, Wolf was Heisenberg’s right hand responsible for designing all tricky behavioral experiments with fruit flies at the flight simulator. Together with Heisenberg, Wolf published six highly cited papers on visually controlled flight orientation in *Drosophila* and optomotor behavior in JCPA.

**Hong Young Yan** (1951-, Taiwan) received his BS (1974) and MS (1979) with work on marine fish at the National Taiwan University. He continued his work on fish as a junior research scientist and assistant research scientist at the Taiwan Fisheries Research Institute and at the Zoological Institute of the Academia Sinica, respectively. In 1982, he joined the Department of Zoology at the University of Texas Austin, where he worked as assistant lecturer and did his doctoral work in the lab of Clark Hubbs on the reproductive strategy of the Clear Creek gambusia. Yan was awarded the Ph.D. in 1986. From 1989–92, he received postdoctoral training on many different topics in marine biology, including on the neuroethology of visual pursuit behavior of mantis shrimp with Thomas W. Cronin* at the University of Maryland Baltimore, and auditory neurophysiology of fish with Arthur N. Popper at the University of Maryland. In 1992, Yan was appointed as adjunct research assistant professor at the University of Kentucky, where he became assistant professor in 1993 and associate professor in 1999. In 2005, he moved back to Taiwan, where he held joint appointments as full professor at the National Taiwan University, the National Taiwan Ocean University, the National I-Lan University and the National Sun Yat Sen University. Presently, he is senior research fellow in the Sensory Physiology Laboratory at the Marine Research Station Institute of Cellular and Organismic Biology in Academia Sinica, Taiwan. He was awarded several honors such as the Great Teacher Award from the University of Kentucky, the National Science Council Distinguished Scientist Award, and an Advanced Fellowship from the *Hanse-Wissenschaftskolleg* (Institute for Advanced Study) in Delmenhorst, Germany. Yan contributed significantly to the understanding of the comparative sensory physiology and behavior of aquatic animals, in particular to the auditory and visual neuroethology of fish. In his seven articles published in JCPA, he characterized the auditory sensitivity and intensity discrimination ability of cichlid fish, the general hearing ability in fish, with an emphasis of the role of the swimbladder and the suprabranchial chamber in fish audition, and the acoustic communication of freshwater gobies.

**Jochen Zeil** (1949-) was born in Gauting, Germany. He is emeritus professor of ecological neuroscience at the School of Biology of Australian National University. After studying biology at the University of Tübingen, he received his doctorate (*Dr. rer. nat.*) in 1981 on sexual dimorphism in the visual system of flies, supervised by Deszö Varjú (University of Tübingen) and Michael Land* (University of Sussex). Following postdoctoral positions in Germany and Australia, and an associate professorship at Kuwait University in 1993, he joined the Visual Sciences Group at Australian National University in 1995 and became an independent group leader of visual ecology focusing on visual behavior in crabs and bumblebees. Zeil has contributed significantly to understanding visual image processing and mechanisms of visual navigation and memory in ants, crab, wasps, and other organisms. His research is particularly characterized by close attention to the natural visual scenes the animals are experiencing. Twenty-three of his papers, including several highly regarded review articles, have been published in JCPA.

**Günther K.H. Zupanc** (1958-, born in Augsburg, Germany) started out his career as a science journalist before he earned his undergraduate degrees in biology and physics at the University of Regensburg. In 1982, while still an undergraduate student, he published his first book *Fische und ihr Verhalten* (title of English edition: *Fish and Their Behavior*; Tetra Press). Several other book publications followed, including his most recent textbook *Behavioral Neurobiology: An Integrative Approach* (Oxford University Press, Third Edition, 2019). In 1990, he received his PhD in neurosciences from the University of California San Diego under the mentorship of Walter Heiligenberg*. In 1992, after a stint as a research biologist at the Scripps Institution of Oceanography, he returned to Germany to head a junior research group at the Max Planck Institute for Developmental Biology and to obtain his *Habilitation* in animal physiology at the University of Tübingen. In 1997, he joined the faculty of the University of Manchester, where he progressed to the rank of associate professor. In 2002, he moved back to Germany for appointment to a full professorship at the newly founded International University Bremen. In 2009, he assumed the position of full professor and chair of the Department of Biology at Northeastern University. His research centers around the exploration of neural plasticity in the adult central nervous system of weakly electric fish, with particular emphasis on how structural changes in neurons and glia mediate alterations in behavior. Towards this goal, his laboratory employs a multidisciplinary approach, combining methods from a broad range of biological and non-biological disciplines. A significant part of this work has been published in JPCA, to which he also contributes frequently review articles and editorials. He edited three special issues, *Electric Fish: Model Systems for Neurobiology* (Volume 192, Issue 6, 2006), Integrative and Comparative Neurobiology: In Memoriam of Theodore H. Bullock* (Volume 194, Issue 2, 2008), and the *Centennial Issue* (Volume 210, Issue 2, 2024). Zupanc has been a member of the Editorial Board of JCPA since 2008. In 2022, he assumed the role of editor-in-chief of the Journal.

## Data Availability

All data generated or analyzed for this paper are presented in Table 1, 2, 3.
